# Angiopoietin-like 3 monomers are abundant in human plasma but are unable to inhibit endothelial lipase

**DOI:** 10.1172/jci.insight.197827

**Published:** 2025-10-28

**Authors:** Sydney G. Walker, Yan Q. Chen, Kelli L. Sylvers-Davie, Alex Dou, Eugene Y. Zhen, Yuewei Qian, Yi Wen, Mariam Ehsani, Sydney Smith, Rakshya Thapa, Maxwell J. Mercer, Lucy Langmack, Bharat Raj Bhattarai, Michael Ploug, Robert J. Konrad, Brandon S.J. Davies

**Affiliations:** 1 Department of Biochemistry and Molecular Biology, Fraternal Order of Eagles Diabetes Research Center, and Obesity Research and Education Initiative, University of Iowa, Iowa City, Iowa, USA.; 2 Lilly Research Laboratories, Eli Lilly and Company, Indianapolis, Indiana, USA.; 3 Department of Chemistry, Waldorf University, Forest City, Iowa, USA.; 4 Finsen Laboratory, Copenhagen University Hospital-Rigshospitalet, Copenhagen N, Denmark.; 5 Finsen Laboratory, Biotech Research and Innovation Centre, University of Copenhagen, Copenhagen N, Denmark.

**Keywords:** Endocrinology, Metabolism, Vascular biology, Lipoproteins

## Abstract

Angiopoietin-like 3 (ANGPTL3) is a major regulator of lipoprotein metabolism. ANGPTL3 deficiency results in lower levels of triglycerides, LDL-cholesterol (LDL-C), and HDL-cholesterol (HDL-C), and may protect from cardiovascular disease. ANGPTL3 oligomerizes with ANGPTL8 to inhibit lipoprotein lipase (LPL), the enzyme responsible for plasma triglyceride hydrolysis. Independently of ANGPTL8, oligomers of ANGPTL3 can inhibit endothelial lipase (EL), which regulates circulating HDL-C and LDL-C levels through the hydrolysis of lipoprotein phospholipids. The N-terminal region of ANGPTL3 is necessary for both oligomerization and lipase inhibition. However, our understanding of the specific residues that contribute to these functions is incomplete. In this study, we performed mutagenesis of the N-terminal region to identify residues important for EL inhibition and oligomerization. We also assessed the presence of different ANGPTL3 species in human plasma. We identified a motif important for lipase inhibition, and protein structure prediction suggested that this region interacted directly with EL. We also found that recombinant ANGPTL3 formed a homotrimer and was unable to inhibit EL activity when trimerization was disrupted. Surprisingly, we observed that human plasma contained more monomeric ANGPTL3 than trimeric ANGPTL3. An important implication of these findings is that previous correlations between circulating ANGPTL3 and circulating triglyceride-rich lipoproteins need to be revisited.

## Abstract 2

We find that ANGPTL3, a major regulator of lipoproteins, cannot inhibit endothelial lipase as a monomer, yet monomers are more abundant than trimers in circulation.

## Introduction

Low levels of HDL-cholesterol (HDL-C) are associated with an increased risk of developing cardiovascular disease (1–3). The clearance of circulating HDL-C is regulated in part by the activity of endothelial lipase (EL) (4–6), which is expressed and secreted by tissues with high metabolic rates such as the liver, lung, and kidneys (4, 7). EL hydrolyzes the phospholipids on the surface of HDL, and this remodeling of the membrane results in lower circulating HDL-C levels (8). Evidence also suggests that EL hydrolyzes the phospholipids of VLDL and VLDL remnant particles, altering their uptake into the liver and the conversion of VLDL remnants to LDL (9).

The activity of EL is inhibited by angiopoietin-like 3 (ANGPTL3) (10). ANGPTL3, which is expressed and secreted by the liver, is a major regulator of lipid homeostasis, inhibiting not only EL, but also lipoprotein lipase (LPL) (10–12). ANGPTL3 deficiency results in lower circulating levels of triglycerides, LDL-cholesterol (LDL-C), and HDL-C through increased activity of EL and LPL (13, 14). Although low HDL-C levels often correlate with an increased risk of cardiovascular disease, individuals deficient in ANGPTL3 are cardio-protected (2, 14, 15). Targeting ANGPTL3 with either antisense oligonucleotides or monoclonal antibodies against ANGPTL3 reduces triglyceride levels in individuals, but the impact of these treatments on cardiovascular disease is not yet known (14, 16). ANGPTL3 must form a complex with the related protein, angiopoietin-like 8 (ANGPTL8), to optimally inhibit LPL (17–20), whereas ANGPTL8 is not required for ANGPTL3-mediated EL inhibition (21). Interestingly, in human serum, total circulating concentrations of ANGPTL3 have been reported to be approximately 10- to 500-fold higher than those of ANGPTL8 (22–26), although it should be noted that not all the methodologies used to measure ANGPTL3 and ANGPTL8 have been fully validated.

ANGPTL3 is a secreted protein that is comprised of an N-terminal domain predicted to form a coiled coil, a linker region that contains a proprotein proteolytic cleavage site, and a C-terminal fibrinogen-like domain (27). The N-terminal region of ANGPTL3 is required for lipase inhibition (12, 27, 28), and within this region, a motif termed the SE1 region (amino acids 32–55) is thought to be especially critical for ANGPTL3 function (29, 30). The N-terminal coiled-coil domain is also essential for ANGPTL3 oligomerization (12), and previous studies have shown ANGPTL3 to form homotrimers and homohexamers (28, 31), although the impact of ANGPTL3 oligomerization on its function is not yet known.

In this study, we utilized alanine-scanning mutagenesis to further understand the contributions of the residues in the N-terminal domain to ANGPTL3 function. To do this, residues in the N-terminal region were mutated two at a time to alanine and analyzed for expression, secretion, oligomerization, and the ability to inhibit EL. In addition, we characterized the oligomeric state of circulating ANGPTL3 by using multiple dedicated immunoassays to analyze size exclusion chromatography (SEC) fractions obtained from human serum.

## Results

### Mutant ANGPTL3 expression.

To identify the residues of ANGPTL3 important for EL inhibition, we performed alanine-scanning mutagenesis of the coiled-coil region of ANGPTL3. Non-alanine residues were mutated in pairs across the N-terminal region of mouse ANGPTL3 from the end of the signal peptide to the cleavage site (residues 17–221), resulting in 99 mutant constructs. Following transfection into HEK293T cells, collection of conditioned media, SDS-PAGE gel electrophoresis, and immunoblotting, we found that the majority of the ANGPTL3 mutants were expressed and secreted at levels comparable to that of wild-type (Supplemental Figure 1; supplemental material available online with this article; https://doi.org/10.1172/jci.insight.197827DS1). Notably, 3 of the ANGPTL3 mutants (M39A-L40A, L120A-N121A, and S166A-F167A) produced no secreted protein after transfection. RT-qPCR analysis indicated that each of these mutant constructs produced mRNA (Supplemental Figure 2, A and B); however, no protein was detected in either the conditioned media or the cell lysate (Supplemental Figure 2C).

### ANGPTL3 cleavage and glycosylation sites.

**Figure 1:**
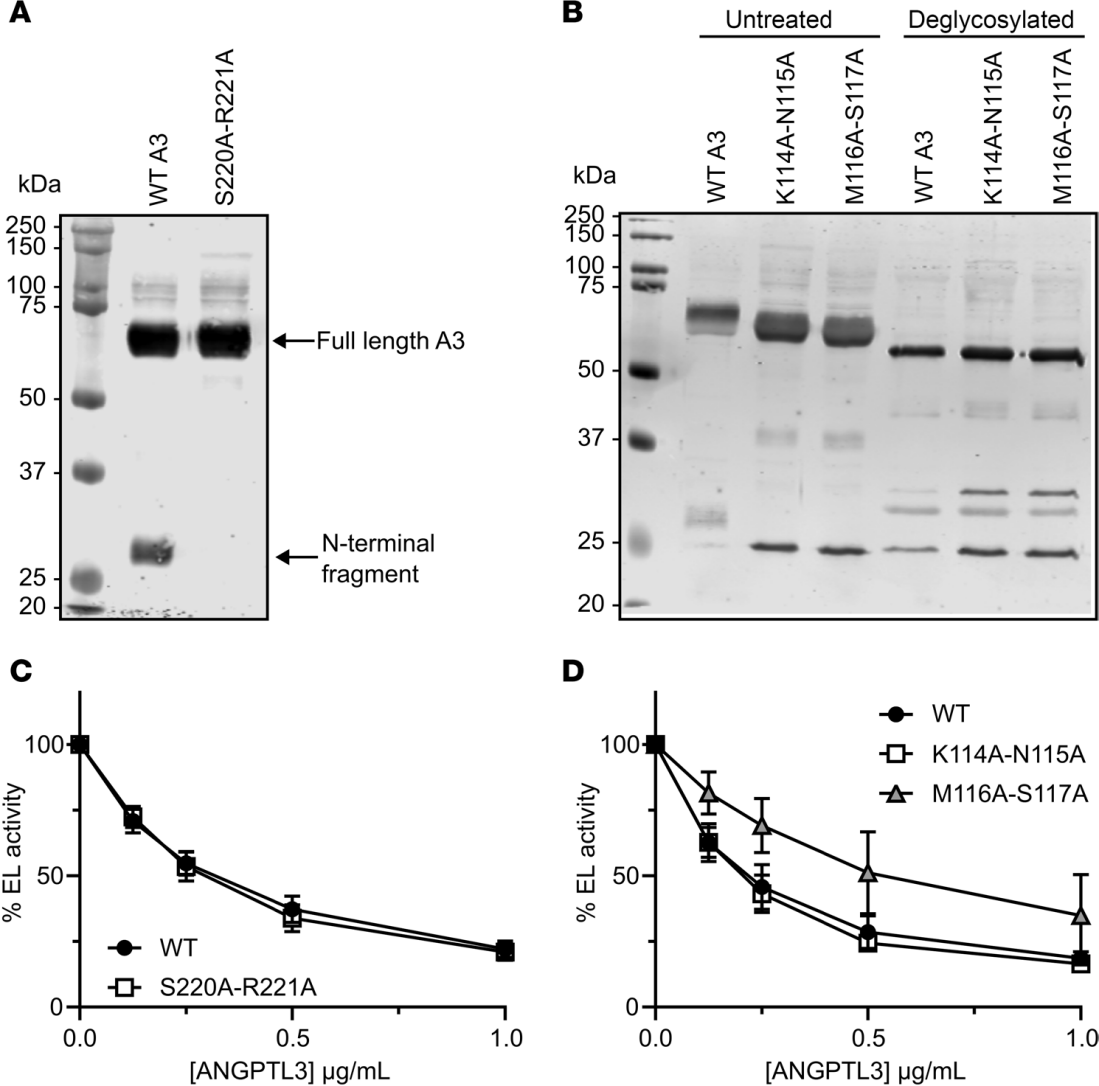
Identification of cleavage-resistant and glycosylation-resistant ANGPTL3 mutations. (**A**) Western blot of conditioned media from cells expressing wild-type ANGPTL3 (WT A3) or ANGPTL3 S220A-R221A. (**B**) Conditioned media from cells expressing WT or the indicated mutant ANGPTL3 were treated with deglycosylation enzymes. The treated and untreated media were analyzed by Western blotting using an antibody against ANGPTL3. (**C** and **D**) The phospholipase activity of EL after incubation with the indicated concentrations of mutant and WT ANGPTL3 for 30 minutes at 37°C was measured using a fluorescence-based phospholipase assay. Data points represent the mean ± SD of 3 independent experiments performed in duplicate. For each graph, the activity was normalized to the control treated with EL and 0 μg/mL ANGPTL3. The experiments shown in panels **C** and **D** were run simultaneously and thus used the same WT control.

Immunoblotting of the conditioned media of the remaining 96 mutants revealed distinct protein properties. Both full-length and cleaved ANGPTL3 were present in wild-type ANGPTL3 and all double-alanine mutants (Supplemental Figure 1) except S220A-R221A, which was resistant to cleavage (Figure 1A). This result aligns well with previous findings showing that ANGPTL3 is cleaved between the N-terminal coiled-coil domain and the C-terminal fibrinogen-like domain at residues 221–225 by proprotein convertases (27, 32–34). ANGPTL3 mutant S220A-R221A removes the protease recognition site and thus was resistant to cleavage.

Although ANGPTL3 mutants K114A-N115A and M116A-S117A were secreted efficiently, the faster electrophoretic migration of both full-length and cleavage fragments with SDS-PAGE suggested that these mutants might have impaired glycosylation (Figure 1B; left side of gel). ANGPTL3 contains an O-linked glycosylation site at Thr226 that has been previously identified as an endogenous regulator of cleavage (35). In N-linked glycosylation, glycans attach to the asparagine amine group at the consensus sequence Asn-X-Ser/Thr, where X represents any amino acid except proline (36). Four N-linked glycosylation motifs are present in the primary sequence of ANGPTL3 at positions 115–117 (^115^Asn-Met-Ser^117^), 232–234 (^232^Asn-Glu-Thr^234^), 296–298 (^296^Asn-Glu-Thr^298^), and 357–359 (^357^Asn-Tyr-Thr^359^). As ANGPTL3 mutants K114A-N115A and M116A-S117A would both eliminate the glycosylation motif at position 115–117 (^115^Asn-Met-Ser^117^), our data strongly suggest that this site is normally utilized. Indeed, when wild-type and mutant proteins were exposed to enzymatic deglycosylation, the resulting fragments now showed similar electrophoretic mobility, confirming that the initial differences in electrophoretic mobility were due to differences in glycosylation (Figure 1B). It should be noted that enzymatic deglycosylation shifted the electrophoretic mobility of full-length protein for both wild-type and mutant constructs (although the shift was larger for wild-type), confirming that ANGPTL3 is glycosylated at multiple sites (Figure 1B). However, deglycosylation did not shift the N-terminal cleavage fragment of the mutant ANGPTL3 proteins, only the wild-type. This demonstrates that glycosylation at Asn115 is the sole glycosylation site utilized in the coiled-coil domain of the protein.

We next determined whether prevention of cleavage or glycosylation of the N-terminal domain affected the ability of ANGPTL3 to inhibit EL. A previous study proposed that cleavage of ANGPTL3 is necessary for its ability to inhibit EL (34). However, we later showed that preventing cleavage of ANGPTL3 in vitro had no effect on its ability to inhibit EL (21). Our present findings are consistent with the idea that cleavage of ANGPTL3 is not required for EL inhibition, as the cleavage-resistant ANGPTL3 (S220A-R221A) inhibited EL similarly to wild-type ANGPTL3 (Figure 1C). Our data also suggest that N-linked glycosylation of the coiled-coil domain of ANGPTL3 is not necessary for EL inhibition, as our ANGPTL3 variant K114A-N115A inhibited EL with the same potency as wild-type ANGPTL3 (Figure 1D). The reason ANGPTL3 M116A-S117A was partially defective in EL inhibition (Figure 1D) remains unexplored, but given the normal inhibitory potency of the K114A-N115A mutant, it is probably not driven by impaired glycosylation.

### Inhibition of EL by mutant ANGPTL3.

**Figure 2:**
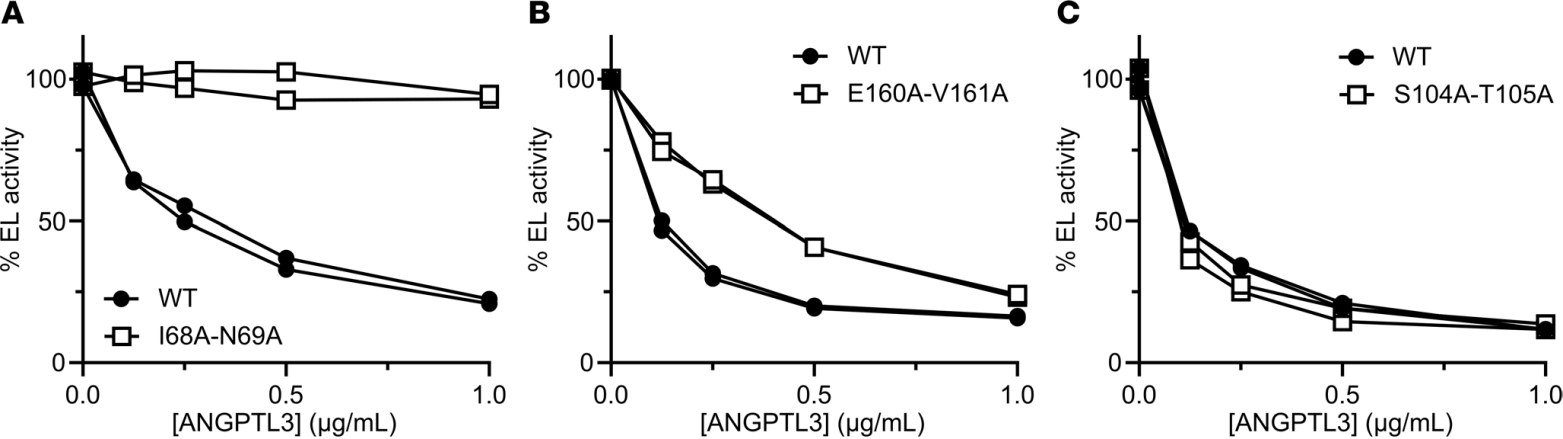
Representative levels of EL inhibition by different ANGPTL3 mutants. Increasing concentrations of wild-type ANGPTL3 (WT) and ANGPTL3 I68A-N69A (**A**), E160A-V161A (**B**), or S104A-T105A (**C**) were incubated with EL for 30 minutes at 37°C. The phospholipase activity of EL was measured using a fluorescence-based phospholipase assay. Points and lines represent the data from a single replicate. For each graph, the activity was normalized to the control treated with EL and 0 μg/mL ANGPTL3.

**Figure 3:**
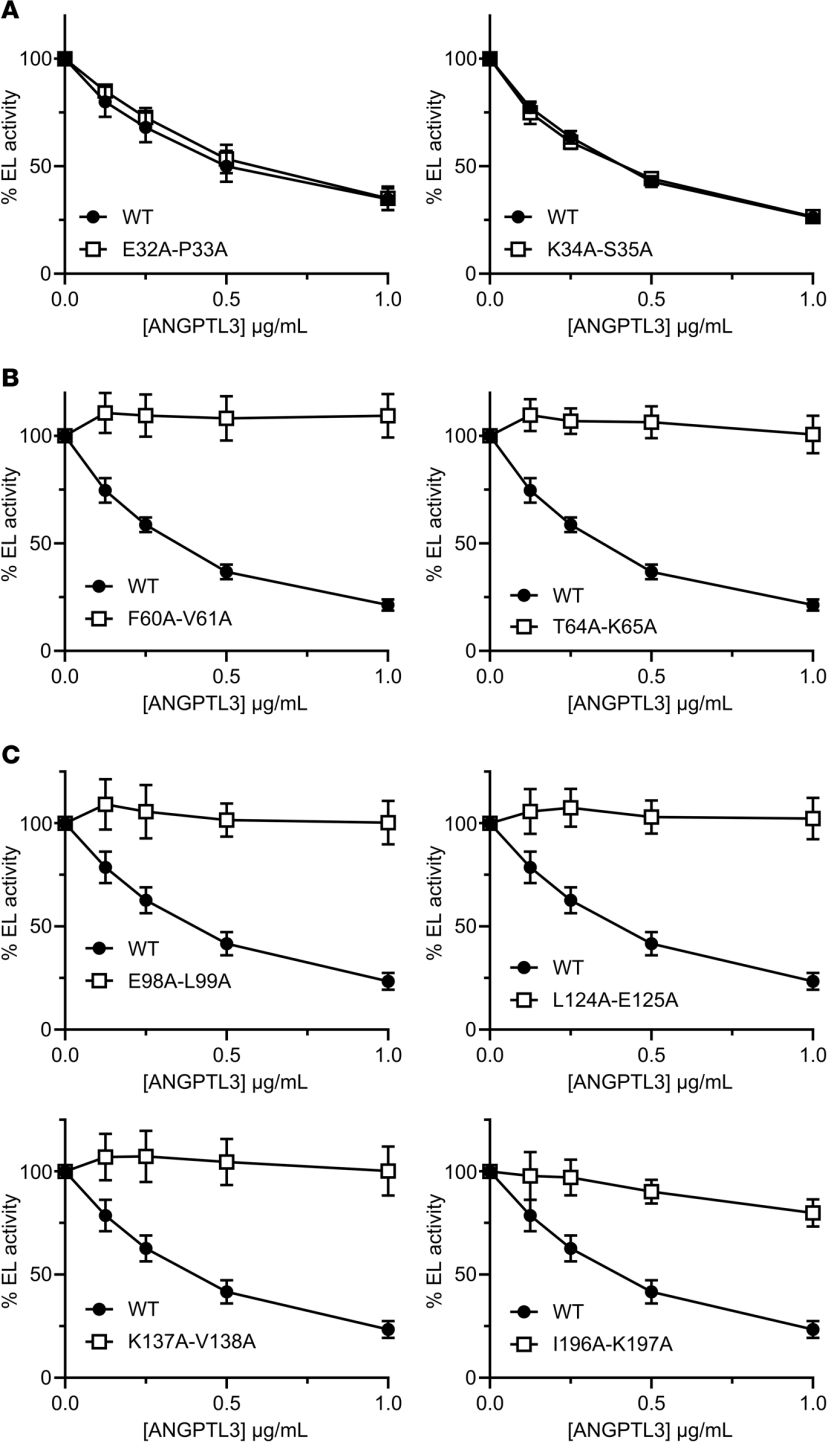
Identification of regions and residues critical for EL inhibition. Phospholipase activity of EL after incubation with the indicated concentrations of wild-type ANGPTL3 (WT) or ANGPTL3 with mutations in the first 4 amino acids of the SE1 motif (**A**), in the region downstream of the SE1 motif (**B**), or in aliphatic hydrophobic residues in the coiled-coil region (**C**). EL and ANGPTL3 were incubated for 30 minutes at 37°C and then phospholipase activity was measured using a fluorescence-based assay. Data points represent the mean ± SD of 3 independent experiments performed in duplicate. For each graph, the activity was normalized to the control treated with EL and 0 μg/mL ANGPTL3. All experiments in panel **B** were performed simultaneously and thus used the same WT control. All experiments in panel **C** were performed simultaneously and thus used the same WT control.

ANGPTL3 is a potent inhibitor of EL, and the main goal of this study was to identify the residues important for this inhibition. Each of the double-alanine ANGPTL3 mutants were tested for their ability to inhibit EL using a fluorescence-based phospholipase A1 assay (Supplemental Table 1 and Supplemental Figure 3). We found the alanine mutations in ANGPTL3 grouped into 3 general classes: mutations that abolished EL inhibition (Figure 2A), mutations that decreased but did not abolish EL inhibition (Figure 2B), and mutations that had no effect on inhibition (Figure 2C). Previous studies have identified that ANGPTL3 contains a highly conserved motif in amino acids 32–55 termed the SE1 region (29, 30). This motif, which is shared with ANGPTL4 and ANGPTL8, is necessary for lipase inhibition and was shown to interact directly with LPL (29, 30, 37). Consistent with this previous finding, most of the mutations in this region resulted in defective EL inhibition (Supplemental Table 1 and Supplemental Figure 3). However, our data suggest that the actual region necessary for lipase inhibition is shifted slightly from the traditional SE1 motif. We found that mutations in amino acids 32–35, the first 4 amino acids of the SE1 motif, had no effect on EL inhibition (Figure 3A), whereas many of the residues in amino acids 56–65, the amino acids C-terminal of the SE1 motif, appeared to be critical for efficient inhibition (Figure 3B). Interestingly, we also found that many residues interspersed throughout the N-terminal coiled-coil domain seemed to be critical for EL inhibition (Figure 3C, Supplemental Table 1, and Supplemental Figure 3). Importantly, for most of the mutants defective in EL inhibition that were outside the SE1 motif, at least one of the mutated residues contained an aliphatic hydrophobic side chain, such as leucine, isoleucine, or valine.

### ANGPTL3 oligomerization.

**Figure 4:**
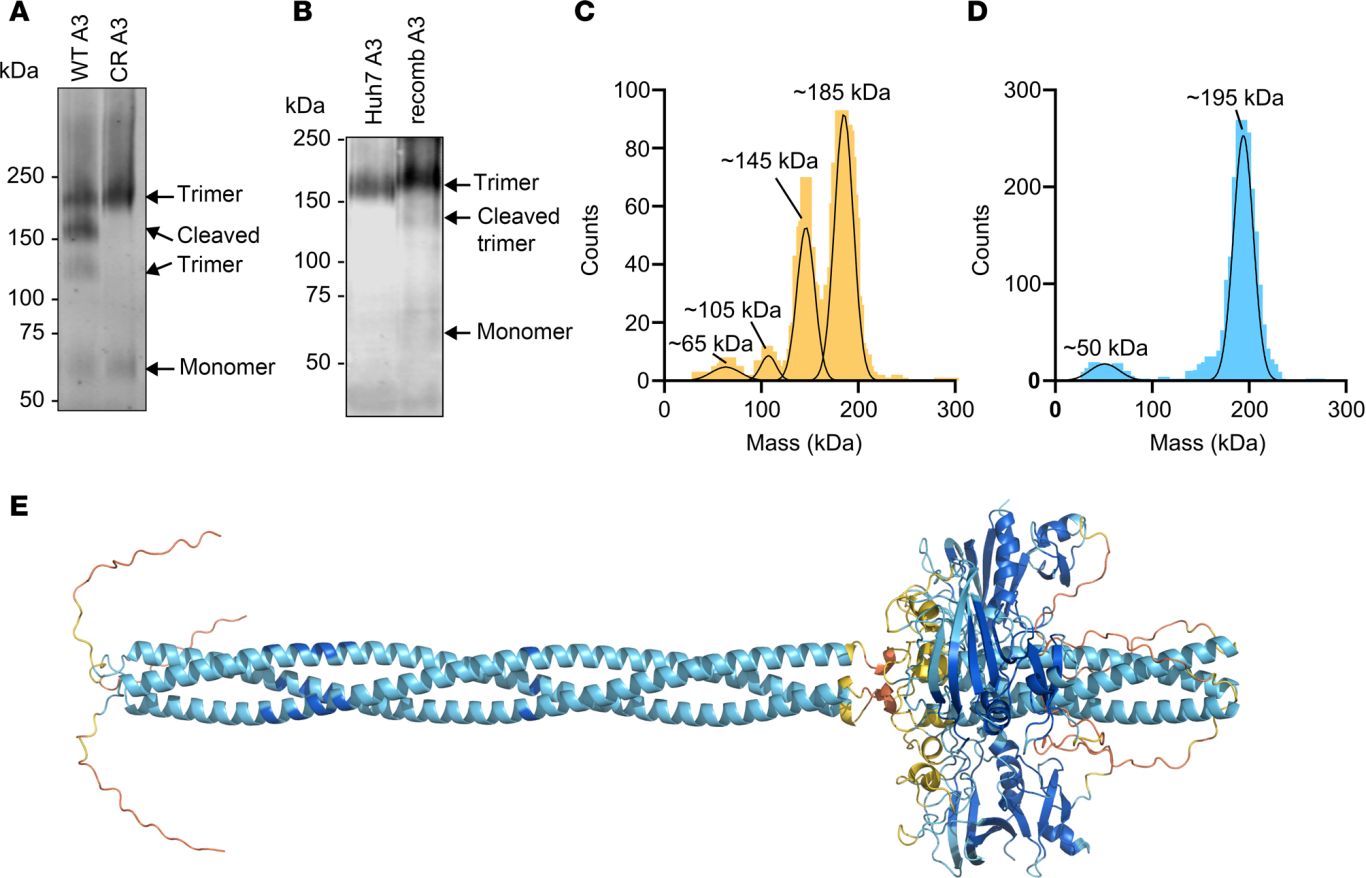
Wild-type ANGPTL3 forms a homotrimer. (**A**) Blue NativePAGE and immunoblotting of recombinant wild-type (WT A3) or cleavage-resistant (CR) ANGPTL3 conditioned media detected with an antibody against ANGPTL3. (**B**) Blue NativePAGE and immunoblotting of endogenous secreted ANGPTL3 from Huh7 cells or recombinant ANGPTL3 (recomb A3) conditioned media detected with an antibody against ANGPTL3. (**C** and **D**) Mass photometry of WT (**C**) or cleavage-resistant (**D**) ANGPTL3 after purification using affinity chromatography. Peaks represent the estimated molecular weight of subpopulations within the sample. (**E**) AlphaFold 3 prediction of the mouse ANGPTL3 homotrimer. Colors indicate predicted local distance difference test (pLDDT) values. Dark blue = very high (pLDDT > 90), light blue = confident (90 > pLDDT > 70), yellow = low (70 > pLDDT > 50), and orange = very low (pLDDT < 50).

We hypothesized that mutations outside of the SE1 motif that affected EL inhibition disrupted ANGPTL3 oligomerization. To test this hypothesis, we first verified the oligomeric state of wild-type ANGPTL3. Previous work reported that ANGPTL3 primarily forms trimers and hexamers as shown by SEC followed by multi-angle light scattering (31) or mass photometry (28). To validate this finding, we utilized blue NativePAGE, a technique that can separate native protein complexes by size and does not require purified proteins (38), followed by immunoblotting for ANGPTL3. We observed a band corresponding to an apparent mass of approximately 190 kDa, which was consistent with the formation of full-length ANGPTL3 homotrimers. We also observed additional bands with slightly faster electrophoretic mobility, which were consistent with homotrimers where 1 or 2 of the C-terminal domains had been cleaved off, and a faster-migrating band consistent with monomers (Figure 4A). To confirm that these additional species were cleavage products, the cleavage-resistant mutant S220A-R221A was analyzed by blue NativePAGE. With this mutant, the intermediate bands were no longer detected, leaving only the major band at approximately 190 kDa and a minor band at approximately 60 kDa, corresponding to a homotrimer and a monomer, respectively (Figure 4A). To ensure that the observed trimeric state of ANGPTL3 was not an artifact of recombinant ANGPTL3 overexpression, we also performed blue NativePAGE on the ANGPTL3 endogenously expressed from human liver–derived Huh7 cells. Here we also observed a dominant band that was consistent with an ANGPTL3 trimer (Figure 4B).

We further validated these findings using purified ANGPTL3 and mass photometry. Wild-type and cleavage-resistant ANGPTL3 were purified from conditioned media using affinity chromatography. Mass photometry, a light-scattering technique that measures the mass of single molecules in solution, was used to assess the oligomeric state of the purified proteins. Consistent with our blue NativePAGE results, most of the purified wild-type ANGPTL3 (58%) was present as a homotrimer at approximately 185 kDa, with additional peaks that were consistent with cleaved homotrimers (~105 kDa and ~145 kDa) and monomers (~65 kDa) (Figure 4C). When the cleavage-resistant ANGPTL3 mutant (S220A-R221A) was analyzed by mass photometry, only a major peak consistent with a homotrimer and a minor peak consistent with a monomer were observed (Figure 4D). Although the structure of the C-terminal fibrinogen-like domain of ANGPTL3 has been previously solved using x-ray crystallography (39), the structure of the full-length ANGPTL3 has not been solved. Given that all our data were consistent with a homotrimer, we used AlphaFold 3 (40) to predict the structure of the ANGPTL3 trimer (Figure 4E). In the predicted structure, the N-terminal domain of ANGPTL3 formed 2 homotrimeric coiled coils separated by an unstructured region. Another unstructured region linked the N-terminal domain and C-terminal fibrinogen-like domain.

The observation that ANGPTL3 forms a trimer coupled with our previous finding that ANGPTL3 and ANGPTL8 form heterotrimers (28) provided an opportunity to reconcile previous discrepancies related to the inhibition of EL by ANGPTL3/8 complexes. Previous studies have reported both that ANGPTL3 and ANGPTL3/8 inhibit EL to a similar degree (21, 41) and that ANGPTL3/8 complexes inhibit EL more potently than ANGPTL3 alone (42). Importantly, the latter study calculated ANGPTL3 and ANGPTL3/8 molarities based on the assumption that ANGPTL3 acted as a monomer and that ANGPTL3/8 complexes consisted of 3 ANGPTL3 molecules and 1 ANGPTL8 molecule (42). When the experiment was repeated, recalculating ANGPTL3 and ANGPTL3/8 molarities based on the finding that both are trimers, ANGPTL3 and ANGPTL3/8 complexes inhibited EL to a similar degree, eliminating the discrepancy between the findings of different groups (Supplemental Figure 4).

**Figure 5:**
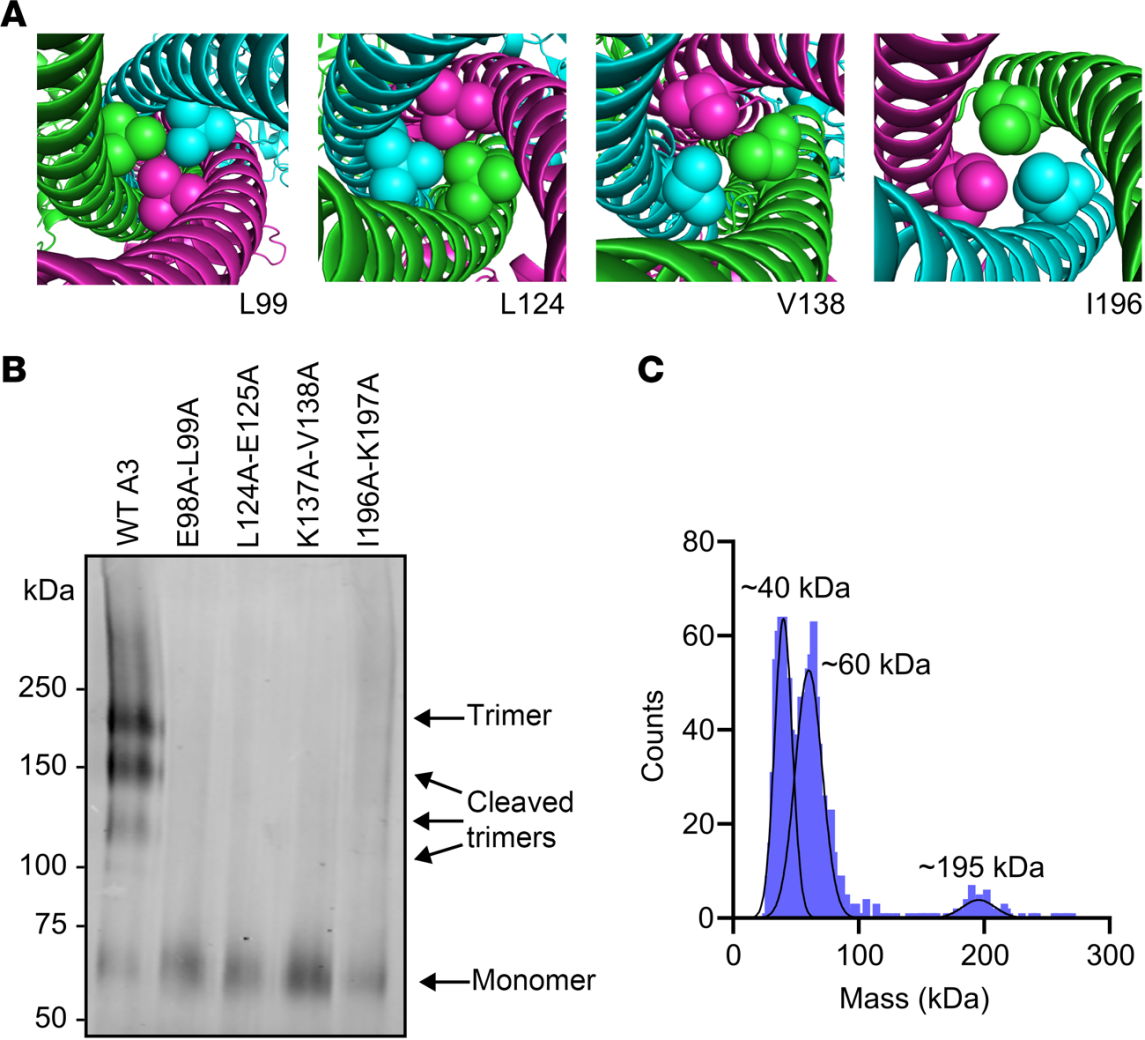
Mutations to aliphatic hydrophobic residues prevent the formation of stable homotrimers. (**A**) AlphaFold 3 prediction of the mouse ANGPTL3 homotrimer showing the indicated residue as spheres at the interface of the 3 ANGPTL3 chains. (**B**) Blue NativePAGE and immunoblotting of wild-type (WT A3) or mutant ANGPTL3 conditioned media detected with an antibody against ANGPTL3. (**C**) Mass photometry of ANGPTL3 mutant L124A-E125A after purification using affinity chromatography. Peaks represent the estimated molecular weight of subpopulations within the sample.

As stated above, for most of the mutants outside the SE1 motif that were defective in EL inhibition, at least one of the mutated residues contained an aliphatic hydrophobic side chain (leucine, isoleucine, or valine). Not surprisingly, when we examined the location of these hydrophobic residues on our AlphaFold-predicted structure, they were located at the interface of the 3 monomers inside the coiled coil (Figure 5A), suggesting that mutation of these residues might disrupt trimer formation. To test our hypothesis, we analyzed wild-type and mutant proteins by blue NativePAGE. We found that most of the mutations within the SE1 motif, although defective in EL inhibition, were not defective in oligomerization (Supplemental Table 1 and Supplemental Figure 5). In contrast, we found that nearly all mutations outside of the SE1 motif that were defective in EL inhibition did not form trimers but presented primarily as a single monomeric band (~60 kDa) after blue NativePAGE (Figure 5B, Supplemental Table 1, and Supplemental Figure 5). To further validate the oligomeric state observed by blue NativePAGE gels, the ANGPTL3 mutant L124A-E125A was purified and analyzed by mass photometry. Two major peaks were observed at 40 kDa and at 60 kDa, corresponding to a C-terminal cleavage product and a monomer, respectively (Figure 5B). The approximately 190 kDa homotrimer peak we had observed with wild-type and cleavage-resistant ANGPTL3 (Figure 4, C and D) had mostly disappeared (Figure 5C), strongly suggesting that this mutant formed very few homotrimers.

**Figure 6:**
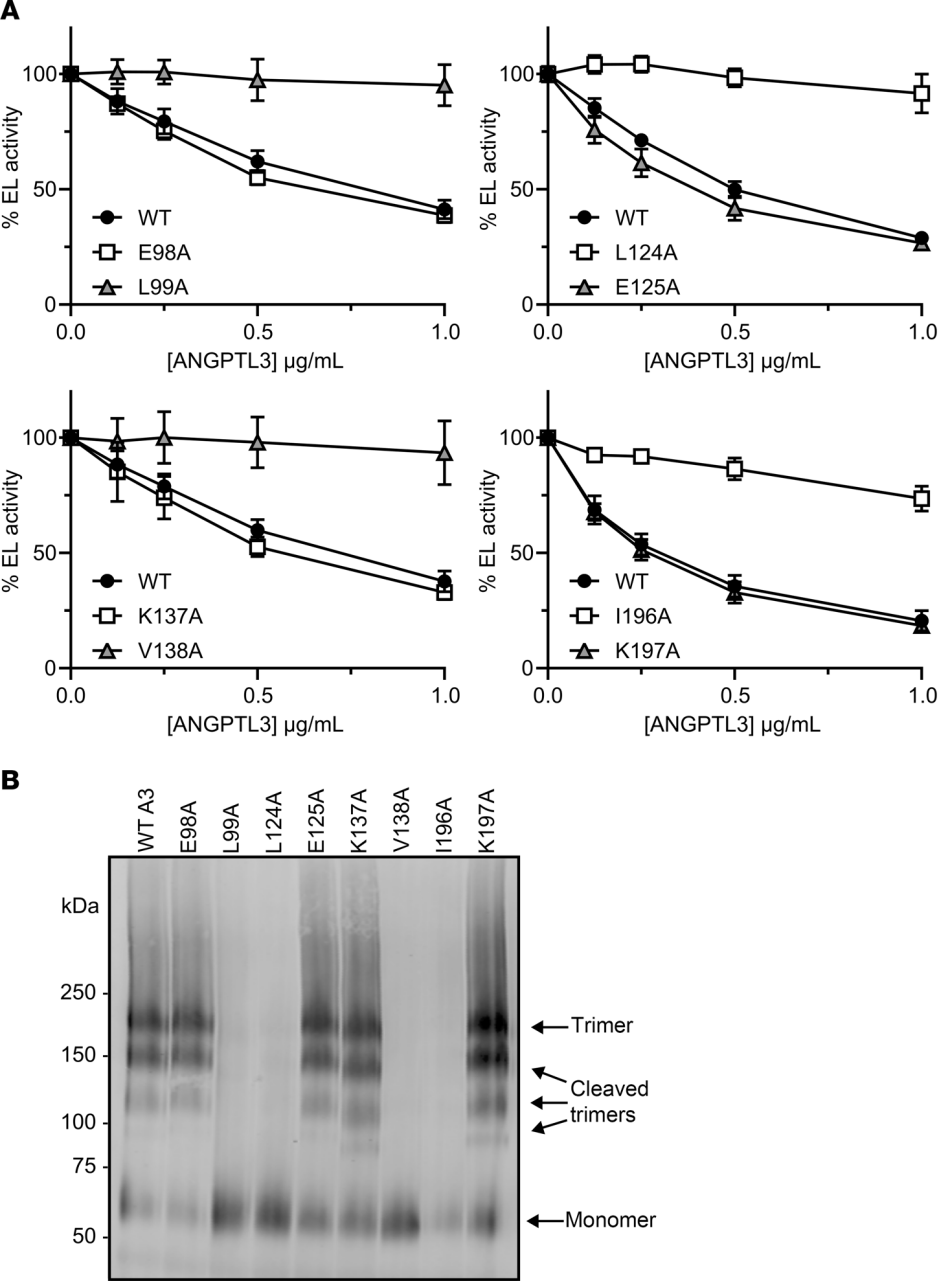
Aliphatic hydrophobic residues are essential for EL inhibition and ANGPTL3 homotrimerization. (**A**) The phospholipase activity of EL after incubation with the indicated concentrations of mutant and wild-type (WT) ANGPTL3 for 30 minutes at 37°C was measured using a fluorescence-based phospholipase assay. Data points represent the mean ± SD of 3 independent experiments performed in duplicate. For each graph, the activity was normalized to the control treated with EL and 0 μg/mL ANGPTL3. The experiments shown in the 2 left-hand graphs were performed simultaneously and thus use the same WT control. (**B**) Blue NativePAGE followed by immunoblotting of WT or mutant ANGPTL3 conditioned media with an antibody against ANGPTL3.

To further validate that it was the mutation of small hydrophobic residues such as leucine, isoleucine, and valine that led to disruption of oligomerization, we chose 4 double mutants that were defective in oligomerization and EL inhibition (E98A-L99A, L124A-E125A, K137A-V138A, and I196A-K197A) and generated the 8 corresponding single mutants. The ability of each of these mutants to oligomerize and inhibit EL was assessed. We found that in each of the paired single mutations, the protein in which the leucine, isoleucine, or valine was mutated lost EL inhibitory function, whereas mutating the neighboring residue did not have any effect on ANGPTL3 function (Figure 6A). Likewise, mutating the leucine, isoleucine, or valine to an alanine disrupted the ability of ANGPTL3 to form homotrimers, but mutating the neighboring residue did not (Figure 6B). These data strongly suggest that these aliphatic hydrophobic side chains are necessary for ANGPTL3 oligomerization. Moreover, across the N-terminal domain, mutants that strongly disrupted trimer formation also strongly impaired EL inhibition (Supplemental Table 1), indicating that trimer formation is necessary for EL inhibition.

Although the focus of this study was the inhibition of EL by ANGPTL3, we also asked whether oligomerization was important for the ability of ANGPTL3 to inhibit LPL. Previous studies have found that ANGPTL3 can inhibit LPL, but that ANGPTL3/8 complexes are much more potent LPL inhibitors (11, 18–20, 43). We tested whether ANGPTL3 D70A-I71A, a mutant that was defective in trimerization and EL inhibition, would also be defective in LPL inhibition. Although, as expected, wild-type ANGPTL3 was not a strong inhibitor of LPL, we found that ANGPTL3 D70A-I71A was even less inhibitory (Supplemental Figure 6A). Moreover, when we expressed this mutant with ANGPTL8, we found that complex formation was compromised (Supplemental Figure 6B) and that there was very little inhibition of LPL by the mutant complex compared with that observed for the wild-type complex (Supplemental Figure 6C).

### ANGPTL3 monomers in human serum.

**Figure 7:**
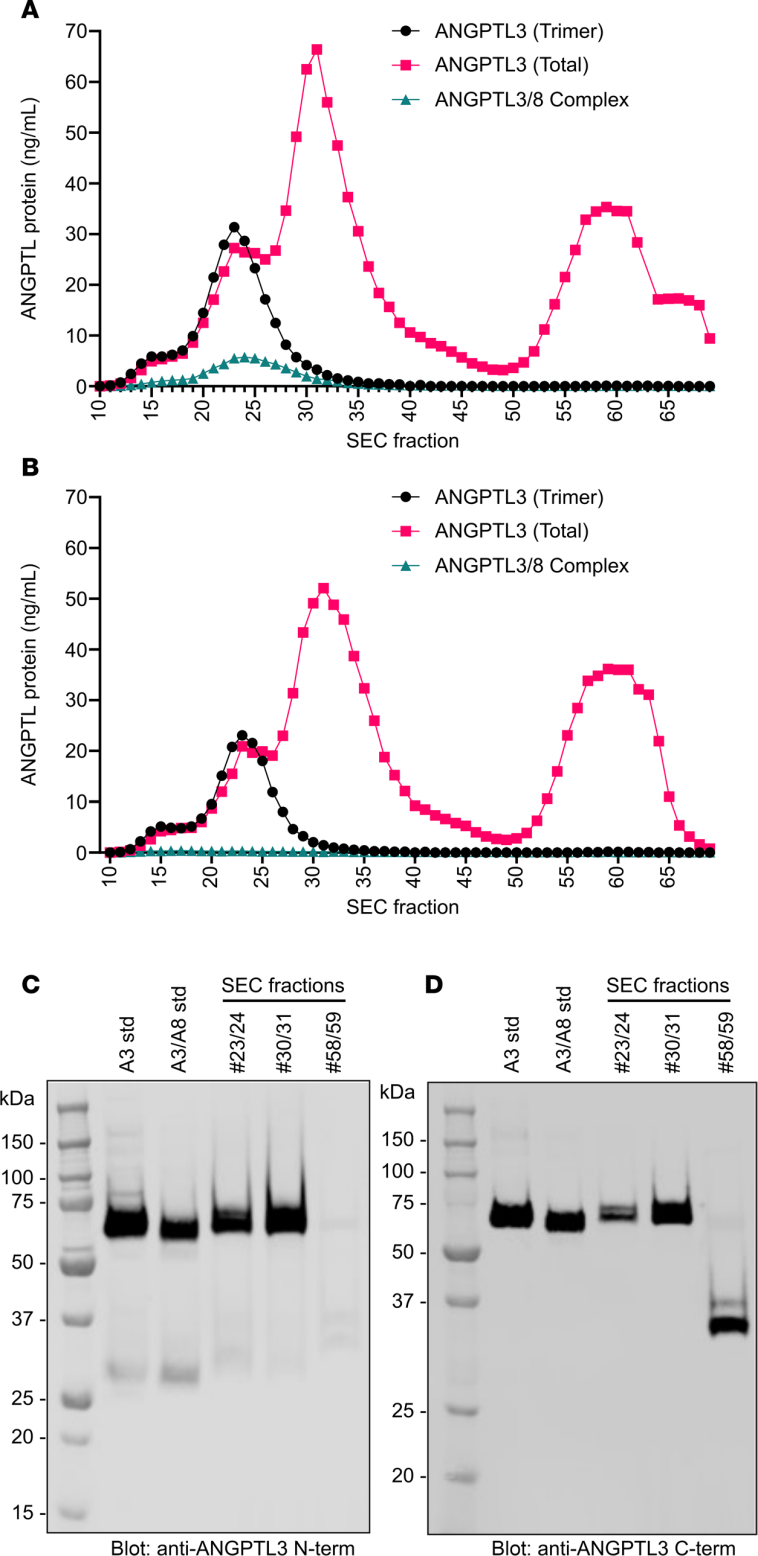
Subpopulations of ANGPTL3 in normal human serum. (**A**) Pooled human serum was fractionated by SEC, and total ANGPTL3, trimeric ANGPTL3, or ANGPTL3/8 complexes in the individual fractions were measured using dedicated immunoassays. (**B**) ANGPTL3/8 in pooled human serum was depleted using an anti-ANGPTL8 antibody (IBA363). The depleted serum was then fractionated by SEC and total ANGPTL3, ANGPTL3 trimers, and ANGPTL3/8 complex levels for each fraction were measured using dedicated immunoassays. (**C** and **D**) Western blot analysis of ANGPTL3 in different serum SEC fractions. Neighboring SEC peak fractions from the 3 major peaks in **B** (peak 1: 23 and 24, peak 2: 30 and 31, peak 3: 58 and 59) were pooled. After immunoprecipitating ANGPTL3, Western blotting was performed using antibodies against the N-terminus (**C**) or C-terminus (**D**) of ANGPTL3.

Given that ANGPTL3 monomers are unable to inhibit EL and that we observed monomers even in our wild-type ANGPTL3–conditioned media, we analyzed the prevalence of monomeric ANGPTL3 in human serum. To do so, pooled human serum was subjected to SEC fractionation. Each fraction was then analyzed by 3 different immunoassays. The first detected only ANGPTL3/8 complexes (20), the second detected only ANGPTL3 trimers, and the final immunoassay detected all ANGPTL3 present in the sample (20). ANGPTL3 trimers were detected in a single, early eluting peak (Figure 7A; black line). ANGPTL3/8 complexes were also detected in a single peak, although as expected, these complexes eluted slightly later than the ANGPTL3 trimers (Figure 7A; cyan line). In contrast, total ANGPTL3 was detected in at least 3 large peaks: one that corresponded to the elution of ANGPTL3 trimers and ANGPTL3/8 complexes, a second, larger overlapping peak that eluted later, and a third peak that eluted much later (Figure 7A; pink line). To further investigate the characteristics of the 3 ANGPTL3 peaks, we depleted serum of ANGPTL3/8 complexes using an ANGPTL8-specific antibody and then subjected the depleted serum to SEC and immunoassays (Figure 7B). We then pooled the 2 fractions with the highest levels of ANGPTL3 for each peak, immunoprecipitated ANGPTL3, and then performed Western blotting using antibodies against both the N-terminal domain and the C-terminal domain of ANGPTL3. Both the first (fractions 23/24) and second (fractions 30/31) ANGPTL3 peaks had mostly full-length ANGPTL3 as indicated by full-size bands when blotted for either the N-terminal or C-terminal domain (Figure 7, C and D). In contrast, the fractions for the third peak (fractions 58/59) had very little signal when blotted with the N-terminal domain antibody (Figure 7C), but a strong signal when blotted with an antibody against the C-terminal region (Figure 7D), indicating that the third peak represented the cleaved, C-terminal domain of ANGPTL3.

**Figure 8:**
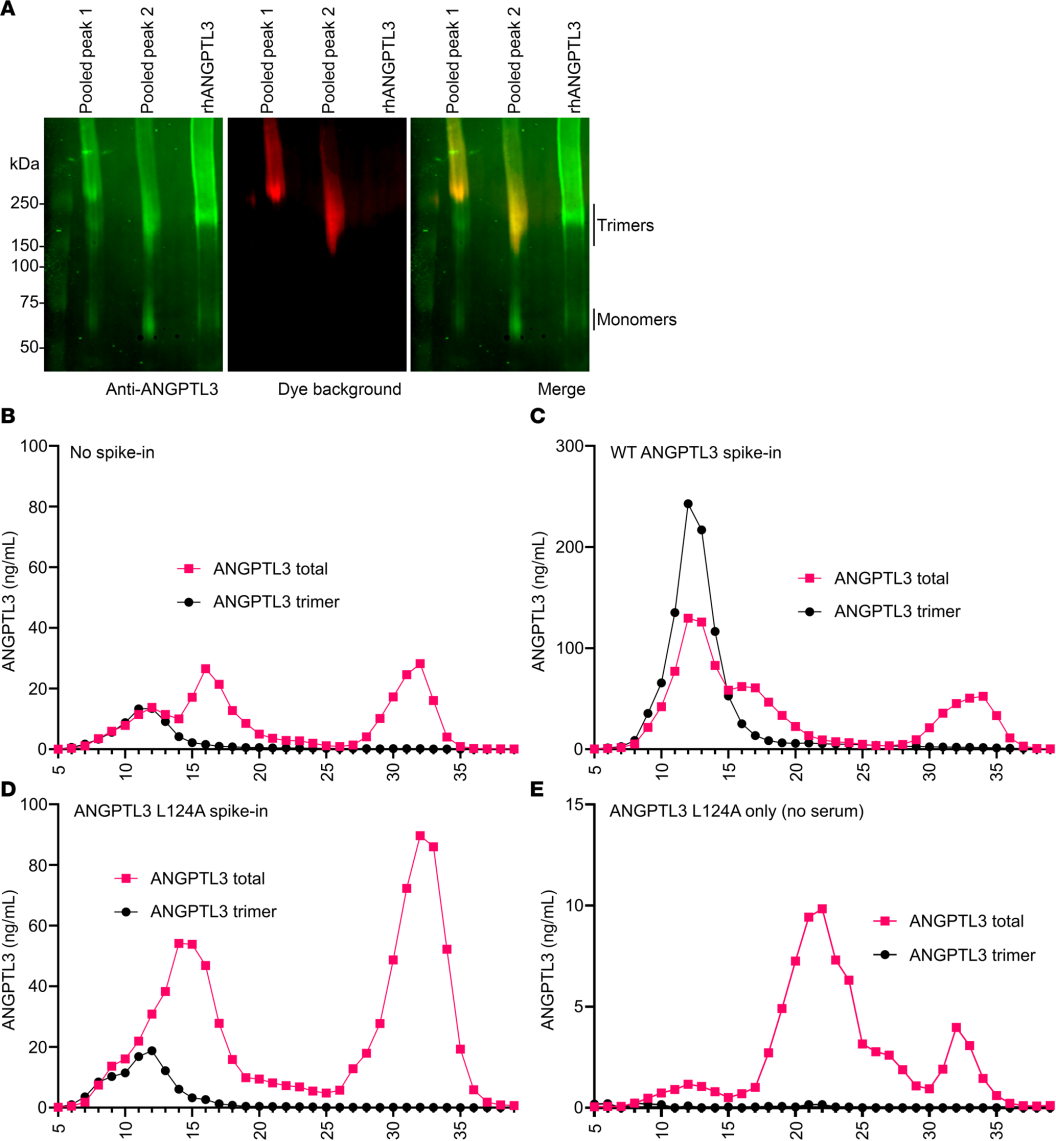
Large amounts of ANGPTL3 monomers are present in human serum. (**A**) Neighboring SEC peak fractions from the first 2 major peaks were pooled and immunoprecipitated for ANGPTL3. ANGPTL3 was then subjected to blue NativePAGE and immunoblotting with an ANGPTL3 antibody. Recombinant human ANGPTL3 (rhANGPTL3) conditioned media served as a control. Red coloring shows the position of non-specific fluorescence from the Coomassie G-250 dye. (**B**–**E**) SEC fractionation of pooled human serum that was run without recombinant protein (**B**) or was spiked with recombinant wild-type (WT) human ANGPTL3 (**C**) or human ANGPTL3 L124A (**D**). Human ANGPTL3 L124A was also run without serum (**E**). Total ANGPTL3 and trimeric ANGPTL3 in the individual fractions were measured using dedicated immunoassays.

The fact that the second peak contained mostly full-length ANGPTL3 that could not be detected by the trimeric ANGPTL3 immunoassay suggested that the fractions within the second peak contained monomeric ANGPTL3. Indeed, blue NativePAGE of immunoprecipitated ANGPTL3 from the pooled fractions from the second peak confirmed the presence of monomeric ANGPTL3 (Figure 8A). For further confirmation, we repeated SEC on the pooled plasma, this time spiking in either recombinant wild-type human ANGPTL3 or human ANGPTL3 with an L124A mutation, which disrupts trimer formation (data shown in Figure 6B). As before, without recombinant ANGPTL3, we observed a single peak when measuring trimeric ANGPTL3 and 3 peaks when measuring total ANGPTL3 (Figure 8B). When human serum was spiked with wild-type trimeric ANGPTL3, we observed a large increase in the ANGPTL3 trimer peak and in the corresponding total ANGPTL3 peak, but little change in the second ANGPTL3 peak (Figure 8C), confirming that the first peak represented trimeric ANGPTL3. Spiking in the L124A mutant ANGPTL3 resulted in little change in the trimeric peak but greatly increased the second peak (Figure 8D), indicating that the second peak represents monomeric ANGPTL3. Together, these data strongly suggest that not only does human plasma contain monomeric ANGPTL3, but that it is the most abundant species and is present at much higher levels than trimeric ANGPTL3.

The origin of monomeric ANGPTL3 in human plasma is not clear. In all our cellular experiments, including those from human liver–derived cells, wild-type ANGPTL3 was predominantly secreted as a trimer (data shown in Figure 4, A and B, Supplemental Figure 5, Figure 5B, and Figure 6B). These data, while not definitive, suggest that the monomeric ANGPTL3 we observed in plasma was secreted as a trimer, but eventually disassociated into monomers. The large number of monomers in circulation also suggest that ANGPTL3 monomers are either unable or are prevented from re-forming trimers. Interestingly, SEC fractionation suggests that there is an abundant serum protein that binds monomeric ANGPTL3. As described above, when we spiked in monomeric L124A mutant ANGPTL3, we saw a large increase in the second ANGPTL3 peak (Figure 8D). However, this second peak eluted sooner (i.e., had a greater apparent molecular weight) than expected for a molecule one-third the size of the trimeric peak. In fact, when we performed SEC on the recombinant L124A mutant ANGPTL3 by itself (rather than spiking it into the serum), we found that it eluted later than it does when spiked into serum (Figure 8E). This strongly suggests that an abundant protein in serum binds to monomeric ANGPTL3, causing it to elute earlier. The identity of this protein, and whether it causes trimer association or simply binds monomers after trimers dissociate is not yet known.

**Figure 9:**
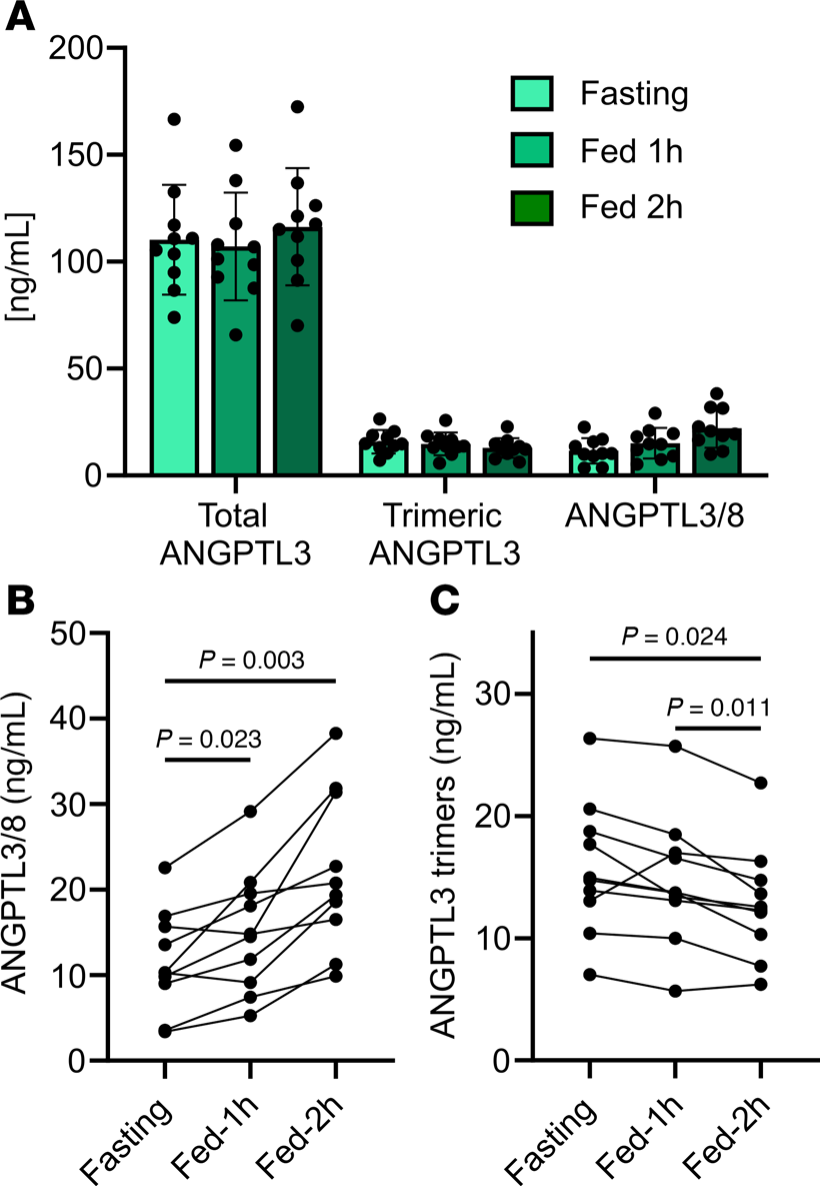
Levels of monomeric and trimeric ANGPTL3 in human serum under different feeding states. (**A**) Total ANGPTL3, trimeric ANGPTL3, and ANGPTL3/8 complex levels as measured by immunoassays in healthy human plasma donors (*n* = 10) after an overnight fast and 1 and 2 hours after a mixed meal. Bars represent mean ± SD. (**B** and **C**) Concentrations of ANGPTL3/8 complexes (**B**) and trimeric ANGPTL3 (**C**) graphed by individual. *P* values were calculated by 1-way repeated-measures ANOVA followed by Tukey’s multiple-comparison test.

The finding that most ANGPTL3 in circulation was monomeric was surprising. To validate this finding and to understand how consistent this finding was across individuals and under different feeding states, we measured total ANGPTL3, trimeric ANGPTL3, and ANGPTL3/8 complexes in healthy individuals (*n* = 10) after an overnight fast, and 1 and 2 hours after a mixed meal. Consistent with our pooled plasma results, total ANGPTL3 levels were much higher than trimeric ANGPTL3 or ANGPTL3/8 levels in all individuals and at all time points (Figure 9A). As expected, given that ANGPTL8 is a feeding-induced protein (44–47), ANGPTL3/8 complex concentrations increased with refeeding (Figure 9B). Interestingly, trimeric ANGPTL3 levels decreased slightly with refeeding (Figure 9C), suggesting that the increase in ANGPTL3/8 heterotrimers might reduce ANGPTL3 trimer levels.

## Discussion

**Figure 10:**
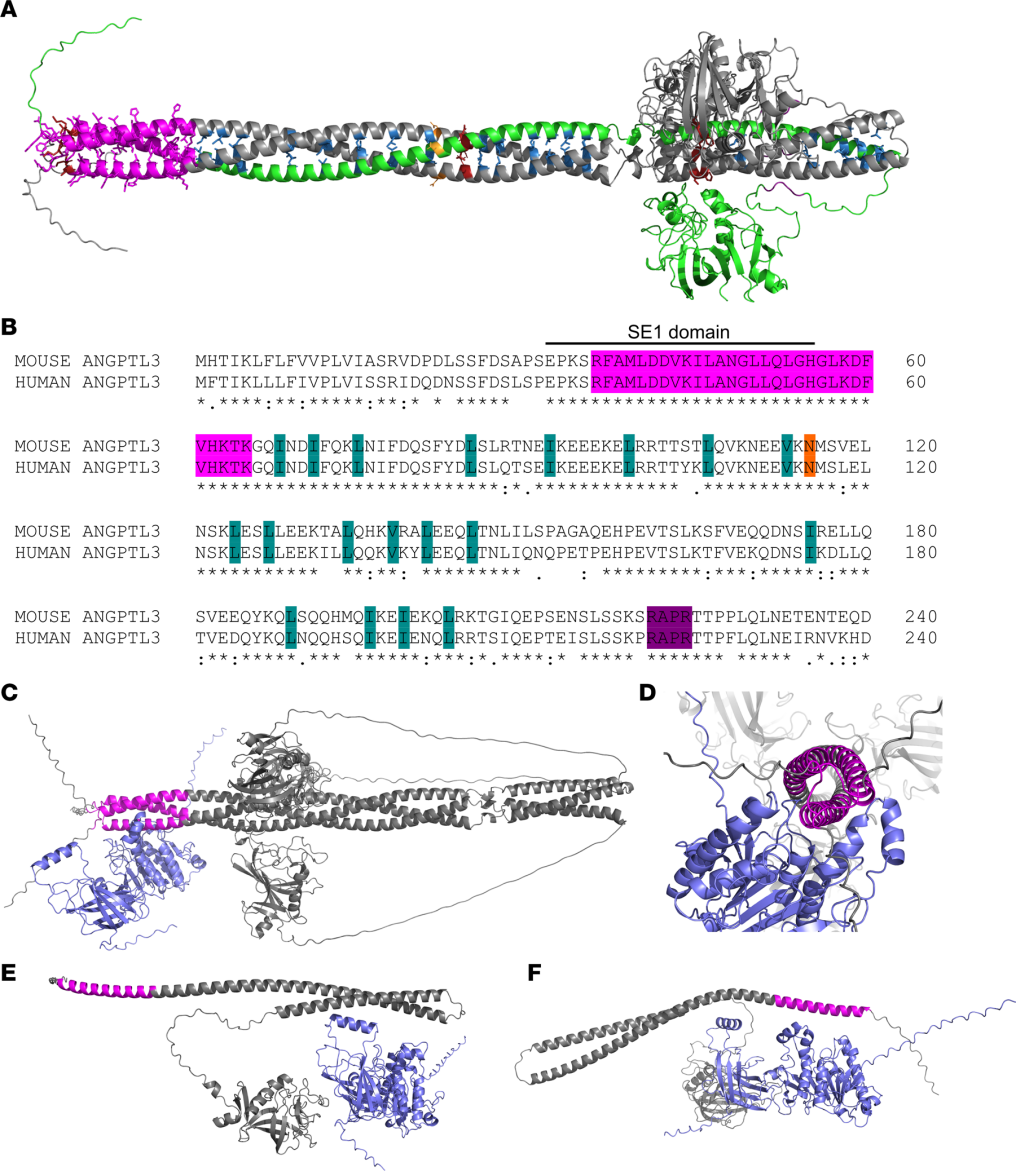
Structure-function models of ANGPTL3. (**A**) Predicted structure of ANGPTL3 showing the lipase interaction domain (magenta), the single glycosylation site in the N-terminal domain (orange), the aliphatic residues necessary for trimer formation, and the cleavage site (purple). The location of mutants that did not express are shown in red and 1 of the 3 ANGPTL3 chains is shown in green. (**B**) Alignment of the N-terminal domains of mouse and human ANGPTL3 using ClustalOmega (62). The lipase interaction domain, glycosylation site, critical trimer residues, and cleavage recognition site are colored as in **A**. The classical SE1 motif is also shown. (**C** and **D**) AlphaFold 3 prediction of the ANGPTL3 homotrimer (gray with the lipase interaction domain in magenta) interacting with EL (slate blue). (**E** and **F**) AlphaFold 3 predictions of an ANGPTL3 monomer interacting with EL.

In this study, we performed site-directed mutagenesis of the N-terminal region of ANGPTL3, identifying residues and motifs important for EL inhibition and ANGPTL3 oligomerization (Figure 10A). We confirmed the proprotein convertase cleavage site of ANGPTL3 and show that the predicted glycosylation site in the coiled-coil domain was indeed glycosylated. We also confirmed that an intact SE1 motif in ANGPTL3 is necessary for EL inhibition, but we also showed that the region critical for lipase inhibition extends further downstream than the previously defined region. We found that wild-type ANGPTL3 forms a homotrimer, that aliphatic hydrophobic side chains along the length of the N-terminal region are critical for ANGPTL3 homotrimerization, and that disruption of ANGPTL3 oligomers abolishes EL inhibition. Surprisingly, we also observed that monomeric ANGPTL3 is abundant in human plasma, with levels far exceeding those of trimeric ANGPTL3.

The N-terminal domain of ANGPTL3 is necessary and sufficient for its inhibition of EL and LPL (10, 27). The N-terminus contains a conserved region termed the SE1 motif (amino acids 32–55), which is critical for binding and inhibiting LPL (29). Previous studies have also shown that certain mutations within this motif abolish EL inhibition (21). Consistent with these findings, most of the mutations we tested in the SE1 motif were defective in EL inhibition. Interestingly, we found mutations in the first 4 amino acids of the SE1 motif (amino acids 32–35) did not affect EL inhibition. However, several mutations downstream of the SE1 motif (amino acids 56–65) greatly reduced the ability of ANGPTL3 to inhibit EL activity. In fact, prior to the characterization of the SE1 domain, Shimamura et al. found that this adjacent region, a putative heparin binding domain, was critical for EL and LPL inhibition (10). Together, these data suggest that the lipase binding and inhibition motif of ANGPTL3 stretches from amino acids 36 to 65, a region completely conserved between mouse and human ANGPTL3 (Figure 10, A and B). Indeed, when the ANGPTL3 trimer was modeled together with EL using AlphaFold, we found that this region is predicted to interact directly with EL (Figure 10, C and D). Although only a prediction, it is important to point out the modeled interaction between the ANGPTL3 trimer and EL shows the SE1 motif nestling into the catalytic cleft of the lipase (Figure 10D), closely resembling the predicted interaction of ANGPTL3/8 heterotrimers with LPL (28). Conversely, when ANGPTL3 monomers were modeled with EL, we did not observe consistent interactions between the SE1 domain of ANGPTL3 and the catalytic cleft of EL (Figure 10, E and F).

ANGPTL3 contains a protease cleavage site at amino acids 221–225 and can be cleaved at this site by the proprotein convertases furin and PACE4 (27, 32–34). The cleavage of ANGPTL3 is also regulated by N-acetylgalactosaminyltransferase 2 (GALNT2), which enzymatically adds O-linked glycans near the cleavage site (Thr226), thus altering the proprotein convertase recognition site (35). The importance of ANGPTL3 cleavage is not entirely clear. Our studies and previous studies found that preventing cleavage, by generating cleavage-resistant forms of ANGPTL3, did not decrease the inhibitory effects of ANGPTL3 in vitro (18, 21, 27). However, a cleavage-resistant form of ANGPTL3 appeared to have impaired inhibitory function in vivo (27), suggesting that the cleavage of the fibrinogen-like domain may improve the circulation or endocrine function of ANGPTL3.

In the original study that identified ANGPTL3, the authors used computational analysis to predict that most (amino acids 19–206) of the N-terminal half of ANGPTL3 forms a coiled-coil structure that likely assembles as a trimer (48). Over 20 years later, Gunn et al. used multi-angle light scattering to show that a refolded protein expressed in *E*. *coli* and only encompassing the N-terminal domain of ANGPTL3 formed homotrimers and homohexamers (31). Recently, mass photometry showed that full-length ANGPTL3 expressed in *Drosophila* S2 cells also adopts a trimeric state (28). Our data strongly support the conclusion that ANGPTL3 forms a homotrimer. Protein structure prediction by AlphaFold further suggested that most of the N-terminal region has a coiled-coil structure, consistent with the original prediction. We found that mutation in any one of several aliphatic hydrophobic residues (mostly leucines) throughout the N-terminal region disrupted trimer formation, supporting the idea that the N-terminal region of ANGPTL3 is a leucine zipper–like structure stabilized by hydrophobic interactions.

Mutations in aliphatic residues of leucine zippers can alter the oligomeric state and function of the protein (49, 50). Based on our oligomerization and inhibition studies, both appear to be true for ANGPTL3. Any mutation that disrupted trimer formation also impaired inhibition, and mutations that formed little to no trimers had almost no ability to inhibit EL, strongly supporting the idea that monomeric ANGPTL3 is inactive. Given this correlation, we were surprised to find that in human plasma, monomeric ANGPTL3 was much more abundant than trimeric ANGPTL3, suggesting that most circulating ANGPTL3 is unable to inhibit EL. The implications of this finding are important. Several studies have shown that ANGPTL3/8 complexes are much more effective inhibitors of LPL than ANGPTL3 alone (17, 18). However, previous studies also determined that circulating human ANGPTL3 levels are 10- to 500-fold higher than ANGPTL8 levels (22–26). If all ANGPTL3 protein was inhibition-competent, the higher abundance of ANGPTL3 would compensate for the greater specific activity of ANGPTL3/8 complexes. Our data reveal a large population of monomeric ANGPTL3 in human plasma that is not expected to inhibit lipases. Thus, while our data indicate that ANGPTL3 trimers are more abundant than ANGPTL3/8 complexes, the ratio of ANGPTL3 homotrimers to ANGPTL3/8 heterotrimers is considerably smaller than the ratio of total ANGPTL3 to ANGPTL3/8.

As only ANGPTL3 trimers inhibit EL, the level of trimers versus monomers is likely metabolically important. It will be important to determine how this ratio changes across metabolic and pathological conditions and between individuals. Several previous studies have examined the association of circulating ANGPTL3 levels with various aspects of cardiovascular disease (15, 22, 51–53). However, none of these studies differentiated between monomeric and trimeric ANGPTL3. Our initial data, using immunoassays specific for total and for oligomeric ANGPTL3, suggest that the ratio of total to trimeric ANGPTL3 is relatively stable across healthy individuals and feeding states. However, the levels of these 2 subpopulations have not been measured in pathological conditions.

The origin of ANGPTL3 monomers in plasma remains unclear. These monomers could be derived from homotrimers that have dissociated, but it is also formally possible that some ANGPTL3 could be secreted directly as monomers. Although secreted recombinant and endogenous wild-type ANGPTL3 was primarily trimeric (data shown in Figure 4, A and B, Supplemental Figure 5, Figure 5B, and Figure 6B), it is important to note that the monomeric mutants were efficiently expressed and secreted from cells, suggesting that secretion of ANGPTL3 monomers may be possible. However, it is also possible that the monomeric mutant proteins formed unstable homotrimers intracellularly and then rapidly dissociated after secretion. If circulating monomeric ANGPTL3 originates from dissociated ANGPTL3 trimers, it will be important to identify proteins or other factors that promote the dissociation of trimers or prevent monomers from re-forming trimers, and determine how these factors are regulated. Our SEC data suggest that there is a plasma protein that can efficiently bind monomeric ANGPTL3. Further studies investigating the identity of this protein and its role in regulating ANGPTL3 function are warranted.

Whether circulating monomeric ANGPTL3 has any physiological function remains unclear, but its high abundance in plasma suggests that it may. Although monomeric ANGPTL3 might still affect EL or lipoproteins in some way, it is also possible that monomeric ANGPTL3 has functions unrelated to lipid metabolism. A previous study reported that ANGPTL3 can induce angiogenesis by binding integrins and activating FAK, MAPK, and Akt signaling pathways (54). There are also reports that ANGPTL3 can influence tumor cell progression and metastasis (55, 56). Further investigation will be required to elucidate the function of ANGPTL3 monomers and their relation, if any, to lipoprotein metabolism.

This study is not without limitations, and many unanswered questions remain. One limitation is that none of our monomeric ANGPTL3 mutants were introduced into an animal model. Thus, while our in vitro data overwhelmingly support the idea that monomeric ANGPTL3 is unable to inhibit EL, the situation in vivo has not been directly tested. As discussed above, our study only analyzed pooled human serum and a small number of deidentified individuals. How accurately our data represent the general populace and the degree of individual variation in monomeric ANGPTL3 is unknown. In our mutagenesis studies, we primarily determined the impact of mutating ANGPTL3 residues on EL inhibition and oligomerization. We expect that mutations in the lipase inhibitory region will also reduce LPL inhibition and that the aliphatic residues necessary for ANGPTL3 homotrimerization are also essential for the formation of ANGPTL3/8 heterotrimers, but this concept has not yet been fully tested. We found that ANGPTL3 D70A-I71A, a mutant defective for ANGPTL3 homotrimerization, was also defective for LPL inhibition and heterotrimerization with ANGPTL8. However, it is possible that not all mutations that affect EL inhibition and homotrimerization will affect LPL inhibition and heterotrimerization with ANGPTL8. It is also important to note that while most of the double mutants that disrupted trimerization mutated at least one aliphatic residue, there were a few (e.g., E142A-E143A) where this was not the case. Why these residues are important for trimer formation is not clear.

In summary, our results provide important structural insights into the formation of ANGPTL3 homotrimers and their role in EL inhibition. As therapeutic strategies targeting ANGPTL3 to treat dyslipidemia emerge, it is essential to understand how ANGPTL3 structure relates to function. Although monomeric ANGPTL3 is the most abundant form in human serum, only ANGPTL3 homotrimers can regulate HDL-C by inhibiting EL. In the future it will be critical to understand the function of the large population of monomeric ANGPTL3 present in the circulation.

## Methods

### Sex as a biological variable.

Sex was not considered as a biological variable in this study.

### Production of wild-type and mutant ANGPTL3–conditioned media.

The generation of a plasmid construct expressing Strep-tagged mouse ANGPTL3 (pEB14) has been described previously (18). Using Phusion site-directed mutagenesis (Thermo Fisher Scientific) and pEB14 as a template, we mutated each non-alanine residue in amino acids 17–221 to an alanine. Residues were mutated in pairs such that every mutant construct had 2 adjacent mutations.

To generate wild-type and mutant ANGPTL3 proteins, HEK293T cells (obtained from ATCC) were grown to 80% confluence in complete DMEM supplemented with 5% FBS, 1% L-glutamine, and 1% penicillin/streptomycin. Plasmids were prepared by combining ANGPTL3 DNA with polyethyleneimine (PEI) in OptiMEM (Gibco). The plasmid/PEI mixture was incubated at room temperature and added to cells with antibiotic-free DMEM (5% FBS, 1% L-glutamine). Twenty-four hours after transfection, cell media were changed to serum-free DMEM (1% L-glutamine, 1% penicillin/streptomycin) containing 0.5× ProteaseArrest protease inhibitor cocktail (APExBIO). Seventy-two hours after transfection, cell media containing the secreted ANGPTL3 protein were collected and centrifuged to remove cell debris. The clarified supernatants were used for Western blotting, blue NativePAGE, and EL activity assays as described below. ANGPTL3 concentration in conditioned media was determined by Western blotting using a previously quantified control. To prepare cell lysates, cells were lysed in RIPA buffer containing 1× ProteaseArrest protease inhibitor cocktail. To produce control media, HEK293T cells were transfected and media were collected using the same procedure as above, only no DNA was added to the transfection.

To generate conditioned media containing endogenously expressed and secreted ANGPTL3, Huh7 cells (a gift from Aloysius Klingelhutz, University of Iowa) were grown in complete DMEM supplemented with 5% FBS, 1% GlutaMAX, and 1% penicillin/streptomycin antibiotics. At 80% confluence, media were changed to serum-free DMEM (1% GlutaMAX, 1% penicillin/streptomycin) containing 0.5× ProteaseArrest protease inhibitor cocktail. Forty-eight hours after media change, cell media containing the secreted ANGPTL3 protein were collected and centrifuged to remove cell debris. The clarified supernatants were used for blue NativePAGE as described below.

For the SEC spike-in experiments, wild-type and L124A mutant human ANGPTL3 proteins were generated as previously described (20). L124 was mutated to an alanine in His-tagged human ANGPTL3 by site-directed mutagenesis. Transient transfection of the expression constructs was performed using Fugene6 transfection reagent (Promega) in HEK293T cells. Cell media containing the secreted ANGPTL3 protein were harvested 48 hours after transfection.

### Production of EL-conditioned media.

EL-conditioned media were generated as previously described (21). Briefly, HEK293T cells stably expressing human EL were grown to 80% confluence in DMEM supplemented with 5% FBS, 1% L-glutamine, and 1% penicillin/streptomycin. Media were changed to serum-free OptiMEM (1% L-glutamine, 1% penicillin/streptomycin) containing 1× ProteaseArrest protease inhibitor cocktail and 0.1 U/mL heparin (Fresenius Kabi, LLC). EL-conditioned media were collected after 24 hours. Protein expression was confirmed by Western blotting using a mouse monoclonal antibody against EL (LIPG antibody clone 3C7, Lifespan Biosciences; diluted 1:3000) and activity was tested using an EnzChek Phospholipase A1 assay kit (Invitrogen).

### Production of LPL-conditioned media.

Human LPL was concentrated from the conditioned media of Chinese hamster ovary cells (CHO-K1) stably expressing FLAG-tagged human LPL as previously described (18, 57, 58).

### Western blotting.

Protein samples were fractionated by size in 12% SDS-PAGE gels and then transferred to a nitrocellulose membrane. Membranes were blocked with casein buffer (1% casein, Fisher Science Education) or LI-COR Intercept PBS blocking buffer (LI-COR). Primary antibodies were diluted in casein buffer or LI-COR Intercept PBS blocking buffer with 0.1% Tween 20. Primary antibody dilutions were 1:3000 for a rabbit polyclonal antibody against Strep-tag II (ab76949, Abcam) or 1:3000 for a rabbit polyclonal antibody against ANGPTL3 (PA5-72812, Invitrogen). After washing with PBS-T, membranes were incubated with donkey anti-rabbit Dylight 800 secondary antibody (PISA510044, Invitrogen; 1:6000) diluted in casein or LI-COR Intercept PBS blocking buffer. After washing with PBS-T, antibody binding was detected using an Odyssey Clx Infrared Scanner (LI-COR).

In some cases, conditioned media were treated with deglycosylation enzymes before Western blotting. Wild-type or mutant ANGPTL3–conditioned media were treated with Protein Deglycosylation Mix II (New England Biolabs, P6044S) following the manufacturer’s instructions and analyzed by Western blotting.

### RNA isolation and qPCR analysis.

To assess RNA expression of mutants that did not produce protein, HEK293T cells were transfected with wild-type or mutant ANGPTL3 constructs as described above. After 24 hours, RNA was extracted from the cell monolayer with TRIzol (Invitrogen) using the manufacturer’s instructions. RNA (1 μg) was used to prepare cDNA with a High-Capacity cDNA Reverse Transcription kit (Applied Biosystems) according to the manufacturer’s instructions. Prepared cDNA was used for qPCR analysis using the following primers: human *GAPDH* forward 5′-AATGGGCAGCCGTTAGGAAA-3′, human *GAPDH* reverse 5′-GCGCCCAATACGACCAAATC-3′, mouse *Angptl3* forward 5′-CGACTGCTCTGCCGTTTAT-3′, and mouse *Angptl3* reverse 5′-GCCTGATTGGGTATCACAGTAG-3′. Diluted cDNA, primers, and SYBR Green ER qPCR Supermix reagent (Invitrogen) were combined, and PCR was performed on the QuantStudio 6 Flex system (Applied Biosystems, Iowa Institute of Human Genetics). Gene expression was calculated using the ΔΔCt method (59) with *GAPDH* as the reference gene and normalized to wild-type *Angptl3* expression.

### Lipase activity assays.

Phospholipase activity was measured using the EnzChek Phospholipase A1 assay kit, as described previously (60). EL and ANGPTL3 proteins (final concentrations: 0–1 μg/mL) were incubated together at 37°C for 30 minutes. Following incubation, 50 μL of sample was mixed with 50 μL of substrate solution and were incubated at room temperature (approximately 20°C–22°C) for 30 minutes, reading fluorescence (485 nm excitation/515 nm emission) every 1–2 minutes with an Infinite F200 plate reader (Tecan). Relative phospholipase activity was calculated by determining the slope of the linear part of the curve (typically in the range between 5 to 25 minutes) and then subtracting the slope of the blank (sample with no EL-conditioned media). Points on graphs were plotted as a percentage of the activity of EL incubated without ANGPTL3. To calculate the level of inhibition for each ANGPTL3 mutant compared to wild-type, the area under the curve was calculated for the inhibition curves for wild-type ANGPTL3, mutant ANGPTL3, and a theoretical inhibition curve in which no inhibition occurs. Wild-type inhibition (%) was then calculated using the formula (AUC_no_ _inhibition_ – AUC_mutant_)/(AUC_no_ _inhibition_ – AUC_wild-type_) × 100. Area under the curve was calculated using R studio (https://posit.co/download/rstudio-desktop/). For initial screening of ANGPTL3 mutants (data shown in Figure 3 and Supplemental Table 1), 1–2 experiments with replicate samples were performed. For mutants of particular interest (Figures 1, 3, and 6), 3 independent experiments, each with duplicate samples, were performed.

To compare the ability of ANGPTL3 and ANGPTL3/8 to inhibit EL, we replicated the human EL cell-based assay performed by Chen et al. as previously described (42) with minor modifications. Specifically, based on recent findings (28), the molecular weight of recombinant ANGPTL3/8 was calculated based on it being a heterotrimer (with an ANGPTL3/ANGPTL8 ratio of 2:1), while the molecular weight of recombinant ANGPTL3 was calculated based on it being a homotrimer.

Triglyceride lipase activity was measured using EnzChek Lipase Substrate (Invitrogen) as described previously (18), using a method originally described by Basu et al. (61). LPL and ANGPTL3 or ANGPTL3/8 proteins were incubated together at 37°C for 30 minutes. Following incubation, 50 μL of sample was mixed with 25 μL 4× assay buffer (0.6 M NaCl, 80 mM Tris-HCl pH 8.0, 6% fatty acid–free BSA) and 25 μL of substrate solution (2.48 μM EnzChek Lipase Substrate and 0.01% 3-[*N*,*N*-dimethylmyristylammonio] propanesulfonate zwittergent detergent [Acros Organics]). Samples were then incubated at 37°C for 30 minutes, reading fluorescence (485 nm excitation/515 nm emission) every 1–2 minutes with an Infinite F200 plate reader (Tecan). Relative phospholipase activity was calculated by determining the slope of the linear part of the curve (typically in the range from 5 to 25 minutes) and then subtracting the slope of the blank (sample with no LPL-conditioned media). Points on graphs were plotted as a percentage of the activity of LPL incubated without ANGPTL3.

### Blue NativePAGE and immunoblotting.

Non-denatured protein complexes were separated by size using NativePAGE Bis-Tris 3-12% gels (Invitrogen). Samples were prepared in 1× NativePAGE sample buffer. The upper chamber was filled with prechilled light blue cathode buffer containing 0.002% Coomassie G-250. The lower chamber was filled with prechilled 1× NativePAGE anode running buffer. Gels were run at room temperature and proteins were transferred to PVDF membranes using 1× NuPAGE transfer buffer. After transfer, membranes were washed with 8% acetic acid and air dried. The membranes were reactivated with 100% methanol, blocked in casein or LI-COR Intercept PBS blocking buffer, and incubated with an antibody against ANGPTL3 (1:3000; Invitrogen, PA5-34750) diluted in casein or LI-COR Intercept PBS blocking buffer with 0.1% Tween 20 overnight at 4°C. Membranes were washed with PBS-T and incubated with donkey anti-rabbit Dylight 800 secondary antibody (1:6000; Invitrogen, PISA510044) diluted in casein or LI-COR Intercept PBS blocking buffer. After washing with PBS-T, antibody binding was detected using an Odyssey Clx Infrared Scanner (LI-COR). To calculate percentage trimer formation for each ANGPTL3 mutant, Image Studio (LI-COR) was used to quantify the monomer and trimer bands. The signal from the trimer bands was divided by the signal from all bands, multiplied by 100, and rounded to the nearest 10.

### Affinity chromatography.

To purify ANGPTL3 for mass photometry, a Strep-TactinXT 4Flow (1 mL) column (IBA Lifesciences) was equilibrated with wash buffer (DPBS pH 8, 150 mM NaCl, 1 mM EDTA, 10% glycerol). Filtered conditioned media containing wild-type, cleavage-resistant (S220A-R221A), or mutant (L124A-E125A) ANGPTL3 was loaded onto the column using an NGC Discover Pro FPLC (Bio-Rad) sample pump at 0.5 mL/min. Following a 15 mL wash with wash buffer, samples were eluted with elution buffer (DPBS pH 8, 150 mM NaCl, 1 mM EDTA, 10% glycerol, 25 mM biotin) at a flow rate of 0.5 mL/min while collecting 1 mL fractions. Chromatograms were obtained via a multiwavelength detector set to 215 nm, 260 nm, and 280 nm. The column was regenerated with freshly prepared 20 mM NaOH, washed with water, and stored in 20% ethanol.

### Mass photometry.

Experiments were conducted using a Refeyn TwoMP mass photometer (Refeyn Ltd). Microscope coverslips (24 mm × 50 mm, Thorlabs Inc.) were cleaned by sequential rinsing with Milli-Q water and HPLC-grade isopropanol (Sigma-Aldrich). Silicon gaskets (Grace Bio-Labs) were used to hold the sample drops. All MP measurements were carried out at ambient temperature using DPBS. A protein standard mixture consisting of β-amylase (Sigma-Aldrich, 56, 112, and 224 kDa) and thyroglobulin (Sigma-Aldrich, 670 kDa) was used to calibrate the instrument. Before each measurement, 15 μL of DPBS was used to find focus, which was locked using the default autofocus function. Then, 5 μL of appropriately diluted protein samples was added and mixed briefly before starting movie acquisition. Movies were acquired for 60 seconds (3000 frames) using AcquireMP (version 2.3.0), analyzed with DiscoverMP (version 2.3.0), and plotted using GraphPad Prism (version 10.4.1).

### AlphaFold 3 protein structure prediction.

The structures of a mouse ANGPTL3 homotrimer, a mouse ANGPTL3 homotrimer bound to human EL, and a mouse ANGPTL3 monomer bound to EL were predicted with AlphaFold 3 Server (40), using the protein sequences of mature mouse ANGPTL3 (amino acids 17–455) and mature human EL (amino acids 21–500). The top ranked structures were used for figures. All 5 predicted structures for each combination can be found in the supplemental material as .cif structure files (Supplemental Datasets 1–15).

### SEC of pooled normal human serum.

Gender-pooled human serum obtained from whole blood donated from healthy human participants was purchased from BioIVT and analyzed by SEC using a Superdex 200 column (Cytiva) and the AKTA protein purification system (Cytiva). In some cases, wild-type or ANGPTL3 L124A–conditioned media were spiked into the pooled human serum before SEC. Fractions were analyzed by immunoassays or blue NativePAGE.

### Trimeric ANGPTL3, total ANGPTL3, and ANGPTL3/8 immunoassays.

Total ANGPTL3 concentrations were measured as previously described (20). ANGPTL3/8 concentrations were also measured as previously described (20), except that an ANGPTL3/8-specific antibody was used for capture, and a second ANGPTL3/8-specific antibody recognizing a separate ANGPTL3/8-specific epitope was used for detection. Trimeric ANGPTL3 levels were measured using a MesoScale Discovery (MSD) immunoassay with streptavidin-coated plates in which a biotinylated ANGPTL3 monoclonal antibody (1G12, generated previously; ref. 20) was used for capture and ruthenium-labeled IG12 was used for detection. This assay does not detect monomeric ANGPTL3 or the C-terminal fragment of ANGPTL3.

### Characterization of ANGPTL3 in SEC fractions by Western blotting.

Pooled normal human serum was first depleted of ANGPTL3/8 by using the anti-ANGPTL8 antibody IBA363. The serum was then subjected to SEC fractionation and individual fractions were collected. Selected fractions that contained trimeric ANGPTL3, monomeric ANGPTL3, and the C-terminal fragment of ANGPTL3 were pooled. ANGPTL3 was immunoprecipitated in each of the 3 fractions as described below. Pooled SEC fractions were separated by size in 12% SDS-PAGE gels and transferred onto PVDF membranes. Biotinylated primary antibodies were used that recognize either the N-terminal region (0.2 μg/mL; R&D Systems, AF3829) or C-terminal region (0.2 μg/mL; Invitrogen, PA5-72812) of ANGPTL3. Bands were visualized with Alexa Fluor 680–conjugated streptavidin (1:5000; Invitrogen, S32358) using an Odyssey imaging system (LI-COR).

### ANGPTL3 immunoprecipitation from SEC fractions.

M-280 Tosylactivated Dynabeads (Invitrogen) were conjugated to an anti-ANGPTL3 antibody (1G12, generated previously; ref. 20) following the manufacturer’s instructions and crosslinked using dimethyl pimelimidate (Thermo Fisher Scientific). Human serum SEC fractions were incubated with anti-ANGPTL3 Dynabeads overnight at 4°C and ANGPTL3 complexes were eluted in 1% acetic acid. Complexes were analyzed by Western blotting and blue NativePAGE.

### Mixed-meal tolerance test.

To study fasting and postprandial conditions, sera were obtained from 10 healthy volunteers after overnight fasting and 1 and 2 hours following a mixed-meal breakfast consisting of approximately 400 carbohydrate calories, 400 fat calories, and 100 protein calories.

### Statistics.

Statistics were performed using GraphPad Prism (version 10.6.1). *P* values were calculated by 1-way repeated-measures ANOVA followed by Tukey’s multiple-comparison test. A *P* value of less than 0.05 was considered significant.

### Study approval.

Human serum samples were obtained with written consent from healthy volunteers from the Eli Lilly Research Blood Donor Program and were anonymized to protect the privacy and personal information of the volunteers.

### Data availability statement.

Underlying data for all graphs and charts are available in the Supporting Data Values file.

## Author contributions

SGW, YQC, KLSD, MP, RJK, and BSJD designed research studies. SGW, YQC, KLSD, AD, EYZ, ME, SS, RT, MJM, LL, and BRB conducted experiments. SGW, YQC, KLSD, AD, EYZ, ME, SS, RT, MJM, LL, and BRB acquired data. SGW, YQC, KLSD, AD, EYZ, YQ, YW, RT, MJM, LL, BRB, RJK, and BSJD analyzed data. SGW and BSJD wrote the manuscript.

## Funding support

This work is the result of NIH funding and is subject to the NIH Public Access Policy. Through acceptance of this federal funding, the NIH has been given a right to make the work publicly available in PubMed Central.

- National Heart, Lung, and Blood Institute, NIH grant R01HL162698 (to BSJD).
- American Heart Association grant 24PRE1188556 (to SGW).

## Supplementary Material

Supplemental data

undefined

Unedited blot and gel images

Supporting data values

## Version 1 10/28/2025

In-Press Preview

## Version 2 12/08/2025

Electronic publication

## Version 3 02/03/2026

Typographical error corrected
