# Supplementary material for: Angiopoietin-like 3 monomers are abundant in human plasma but are unable to inhibit endothelial lipase
Source: JCI Insight. 2025 Oct 28;10(23):e197827. doi: 10.1172/jci.insight.197827 (PMC12867014; doi:10.1172/jci.insight.197827)
Supplement: Supplemental data [file jciinsight-10-197827-s265.pdf]

Angiopoietin-like 3 monomers are abundant in human plasma but are unable to inhibit endothelial lipase

Sydney G. Walker[1], Yan Q. Chen[2], Kelli L. Sylvers-Davie[1], Alex Dou[1], Eugene Y. Zhen[2], Yuewei Qian[2], Yi Wen[2], Mariam Ehsani[2], Sydney Smith[2], Rakshya Thapa[1,3], Maxwell J. Mercer[1], Lucy Langmack[1], Bharat Raj Bhattarai[1,3], Michael Ploug[4,5], Robert J. Konrad[2], Brandon S. J. Davies[1,*]

[1] Department of Biochemistry and Molecular Biology, Fraternal Order of Eagles Diabetes Research Center, and Obesity Research and Education Initiative, University of Iowa, Iowa City, IA 52242. [2] Lilly Research Laboratories, Eli Lilly and Company, Indianapolis, IN 46285. [3] Department of Chemistry, Waldorf University, Forest City, IA 50436. [4]Finsen Laboratory, Copenhagen University Hospital-Rigshospitalet, DK-2200 Copenhagen N, Denmark; [5]Finsen Laboratory, Biotech Research and Innovation Centre, University of Copenhagen, DK-2200 Copenhagen N, Denmark

*Address Correspondence to: Brandon S. J. Davies, Department of Biochemistry and Molecular Biology, University of Iowa, 169 Newton Rd., PBDB 3326, Iowa City, IA, 52242. Tel.: 319-335-3225; Fax: 319-335-9570; E-mail: brandon-davies@uiowa.edu.

Supplemental Table 1. Inhibitory activity and trimerization of ANGPTL3 mutants

mutant	% inh	% tri
S17A-R18A	73	100
V19A-D20A	102	100
P21A-D22A	96	100
L23A-S24A	100	90
S25A-F26A	105	100
D27A-D28A	84	90
P30A-S31A	105	100
E32A-P33A	104	90
K34A-S35A	122	100
R36A-F37A	10	80
M39A-L40A	ND	ND
D41A-D42A	26	70
V43A-K44A	36	100
I45A-L46A	11	90
N48A-G49A	52	80
L50A-L51A	-6	60
Q52A-L53A	9	30
G54A-H55A	28	90
G56A-L57A	22	90
K58A-D59A	88	70
F60A-V61A	21	30
H62A-K63A	45	80
T64A-K65A	20	50
G66A-Q67A	86	90
I68A-N69A	4	40
D70A-I71A	3	20
F72A-Q73A	62	40
K74A-L75A	11	40
N76A-I77A	87	100
F78A-D79A	22	60
Q80A-S81A	122	100
F82A-Y83A	5	30
D84A-L85A	11	0

mutant	% inh	% tri
S86A-L87A	95	100
R88A-T89A	87	90
N90A-E91A	91	100
I92A-K93A	6	30
E94A-E95A	119	90
E96A-K97A	149	90
E98A-L99A	-10	10
R100A-R101A	21	50
T102A-T103A	81	90
S104A-T105A	105	100
L106A-Q107A	-2	20
V108A-K109A	107	90
N110A-E111A	92	100
E112A-V113A	-27	10
K114A-N115A	108	100
M116A-S117A	40	60
V118A-E119A	111	90
L120A-N121A	ND	ND
S122A-K123A	56	60
L124A-E125A	6	10
S126A-L127A	39	0
L128A-E129A	91	90
E130A-K131A	27	20
T132A-L134A	8	30
Q135A-H136A	94	90
K137A-V138A	-4	0
R139A-L141A	-2	20
E142A-E143A	11	30
Q144A-L145A	19	30
T146A-N147A	104	90
L148A-I149A	88	70
L150A-S151A	90	100
P152A-G154A	87	90

mutant	% inh	% tri
Q156A-E157A	100	80
H158A-P159A	94	100
E160A-V161A	72	50
T162A-S163A	102	80
L164A-K165A	37	0
S166A-F167A	ND	ND
V168A-E169A	70	60
Q170A-Q171A	88	70
D172A-N173A	108	80
S174A-I175A	30	20
R176A-E177A	103	80
L178A-L179A	54	70
Q180A-S181A	105	80
V182A-E183A	98	90
E184A-Q185A	115	90
Y186A-K187A	100	90
Q188A-L189A	34	0
S190A-Q191A	86	90
Q192A-H193A	76	50
M194A-Q195A	117	90
I196A-K197A	75	50
E198A-I199A	33	20
E200A-K201A	102	80
Q202A-L203A	1	40
R204A-K205A	104	80
T206A-G207A	95	90
I208A-Q209A	99	90
E210A-P211A	97	80
S212A-E213A	96	90
N214A-S215A	92	90
L216A-S217A	116	90
S218A-K219A	112	60
S220A-R221A	100	100

Levels of EL inhibition (% inh) and trimerization (% tri) of each ANGPTL3 mutant as compared to wild-type ANGPTL3. ND = not determined

Supplemental Figure 1

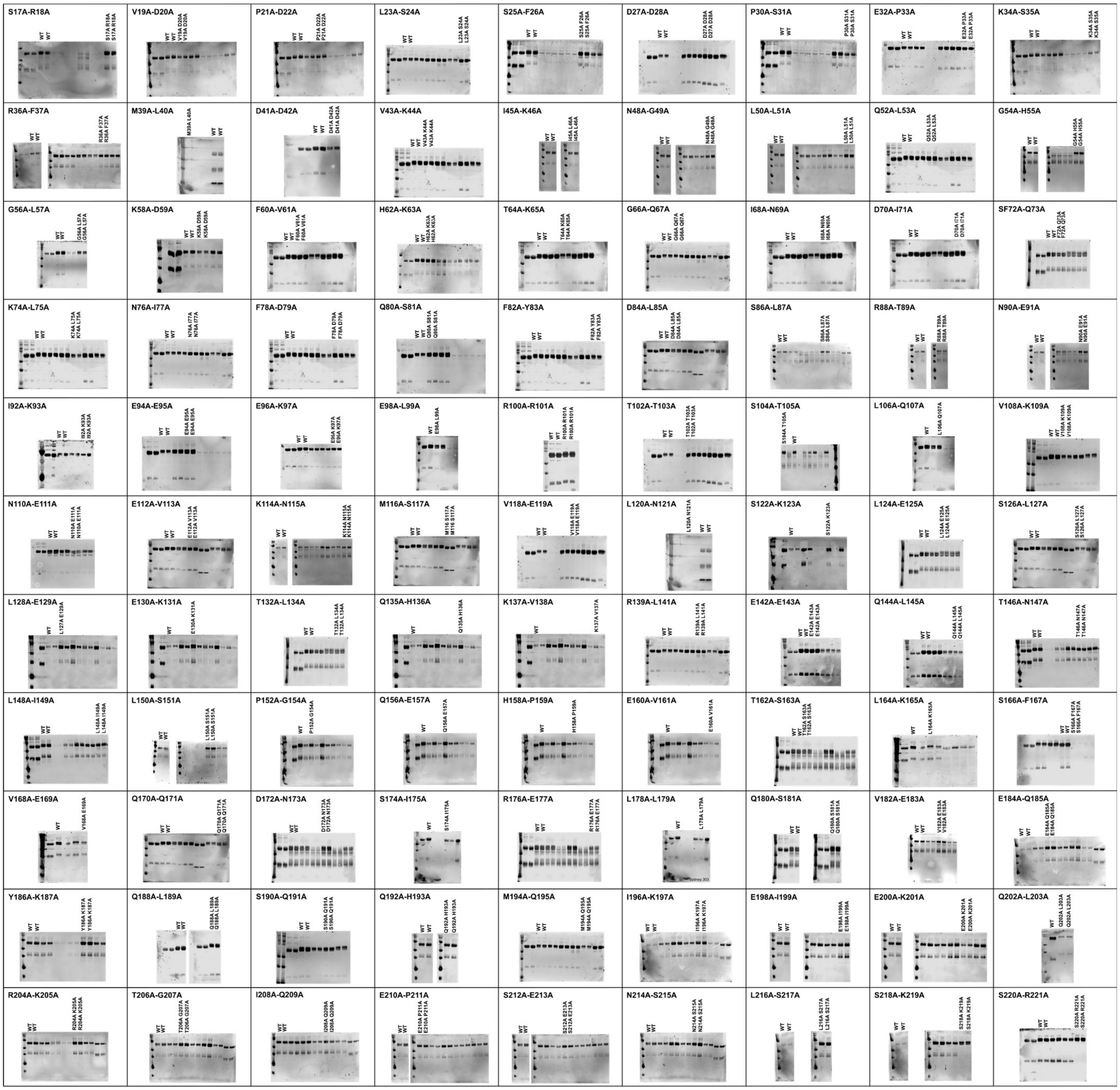

Supplemental Figure 1: Protein expression of ANGPTL3 mutants. Western blots of conditioned media expressing either wild-type or mutant protein.

Supplemental Figure 2

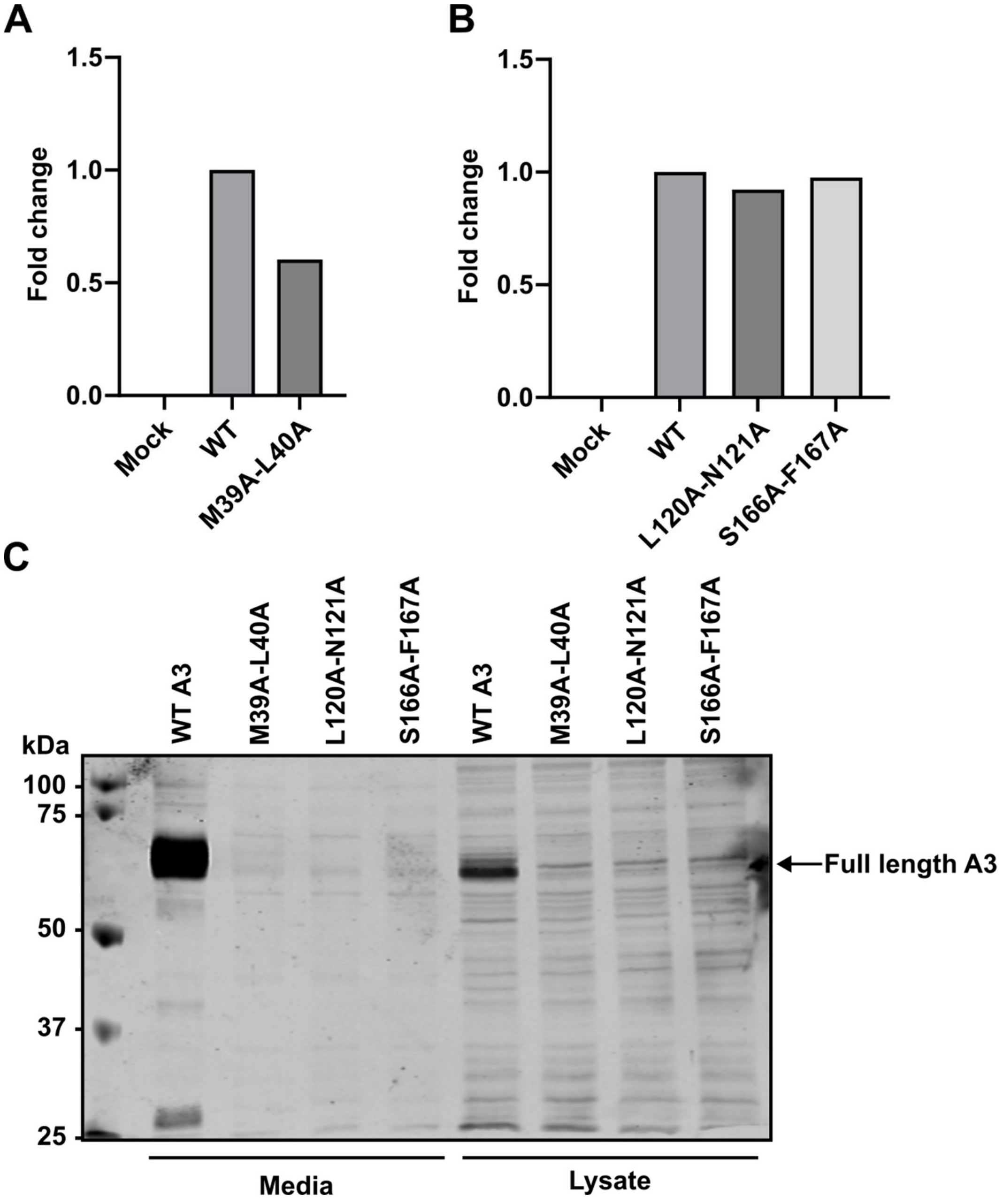

Supplemental Figure 2: ANGPTL3 mutants M39A-L40A, L120A-N121A, and S166A-F167A do not produce viable protein. (A-B) mRNA expression of *Angptl3* in cells transiently transfected with constructs expressing either wild-type or the indicated mutant ANGPTL3 as measured by qPCR. Bars represent the fold change in expression compared to wild-type ANGPTL3-transfected cells. **(C)** Western blot of media and cell lysates from cells transiently transfected with wild-type or the indicated mutant ANGPTL3.

Supplemental Figure 3

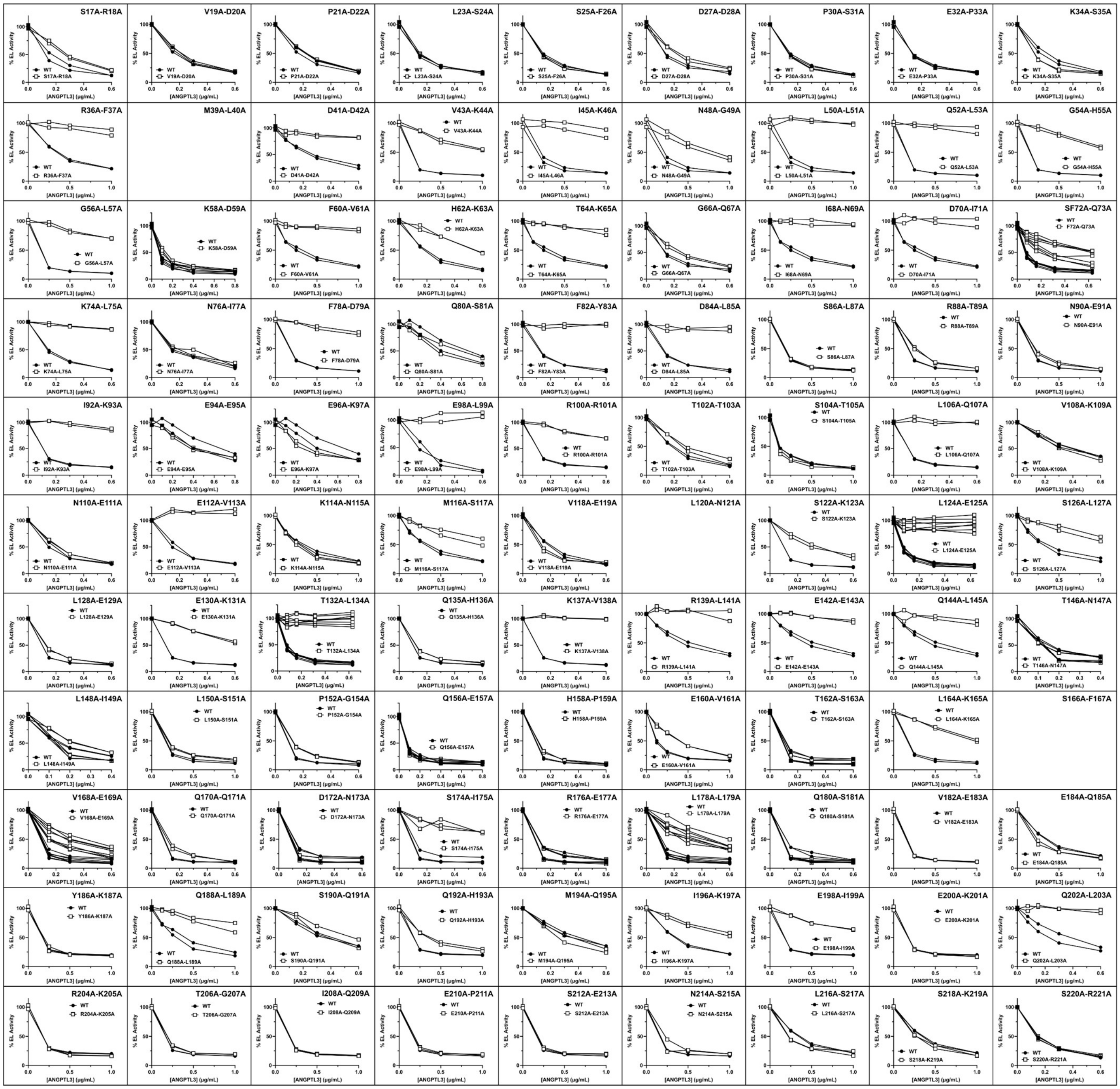

Supplemental Figure 3: EL inhibition by ANGPTL3 mutants. Increasing concentrations of wild-type ANGPTL3 or the indicated ANGPTL3 mutant were incubated with EL for 30 minutes at 37ºC. The phospholipase activity of EL was measured using a fluorescence-based phospholipase assay. Points of each line represent thedata of an individual replicate. For each graph, the activity was normalized to the control treated with EL and 0 µg/ml ANGPTL3. Mutants tested simultaneously used the same WT control.

Supplemental Figure 4

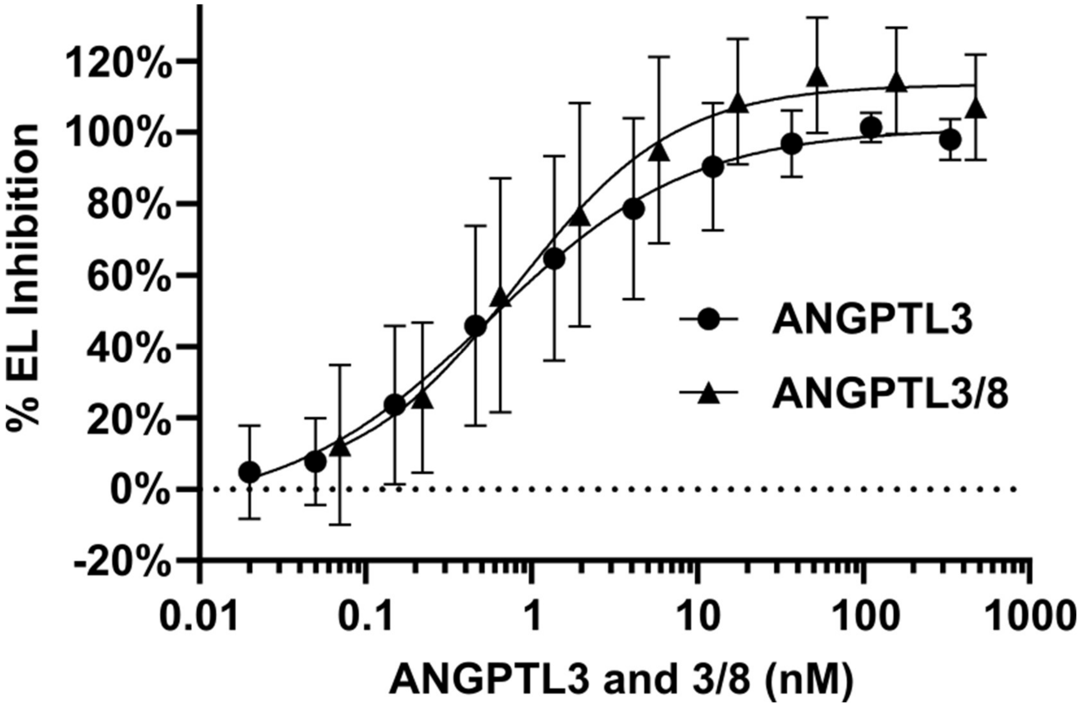

Supplemental Figure 4: ANGPTL3 and ANGPTL3/8 inhibition of EL. The ability of ANGPTL3 or ANGPTL3/8 to inhibit EL at 37°C was assessed using an EL stable expression cell line and phospholipase A1 selective substrate. Fluorescence was monitored with an excitation wavelength of 485 nm and an emission wavelength of 516 nm. Readings were taken at 1 and 30 min, with the 1-minute reading subtracted from the 30-minute reading to correct for background fluorescence. Results are shown as the mean ± SD (n=8-10) from three independent experiments for ANGPTL3 and four independent experiments for ANGPTL3/8.

Supplemental Figure 5

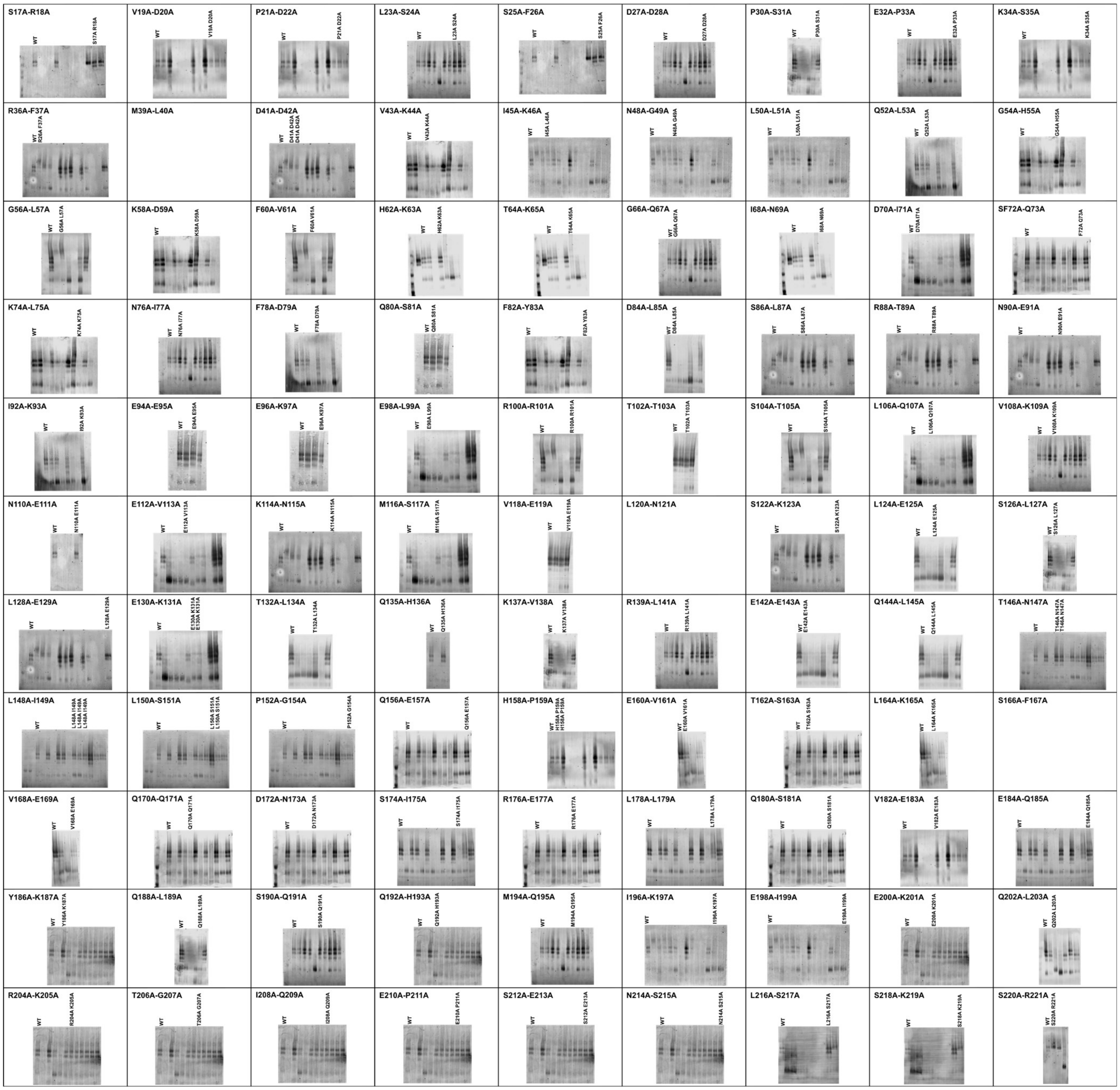

Supplemental Figure 5: Oligomerization of ANGPTL3 mutants. Blue nativePAGE gel electrophoresis followed by immunoblotting of wild-type or the indicated ANGPTL3 mutant.

Supplemental Figure 6

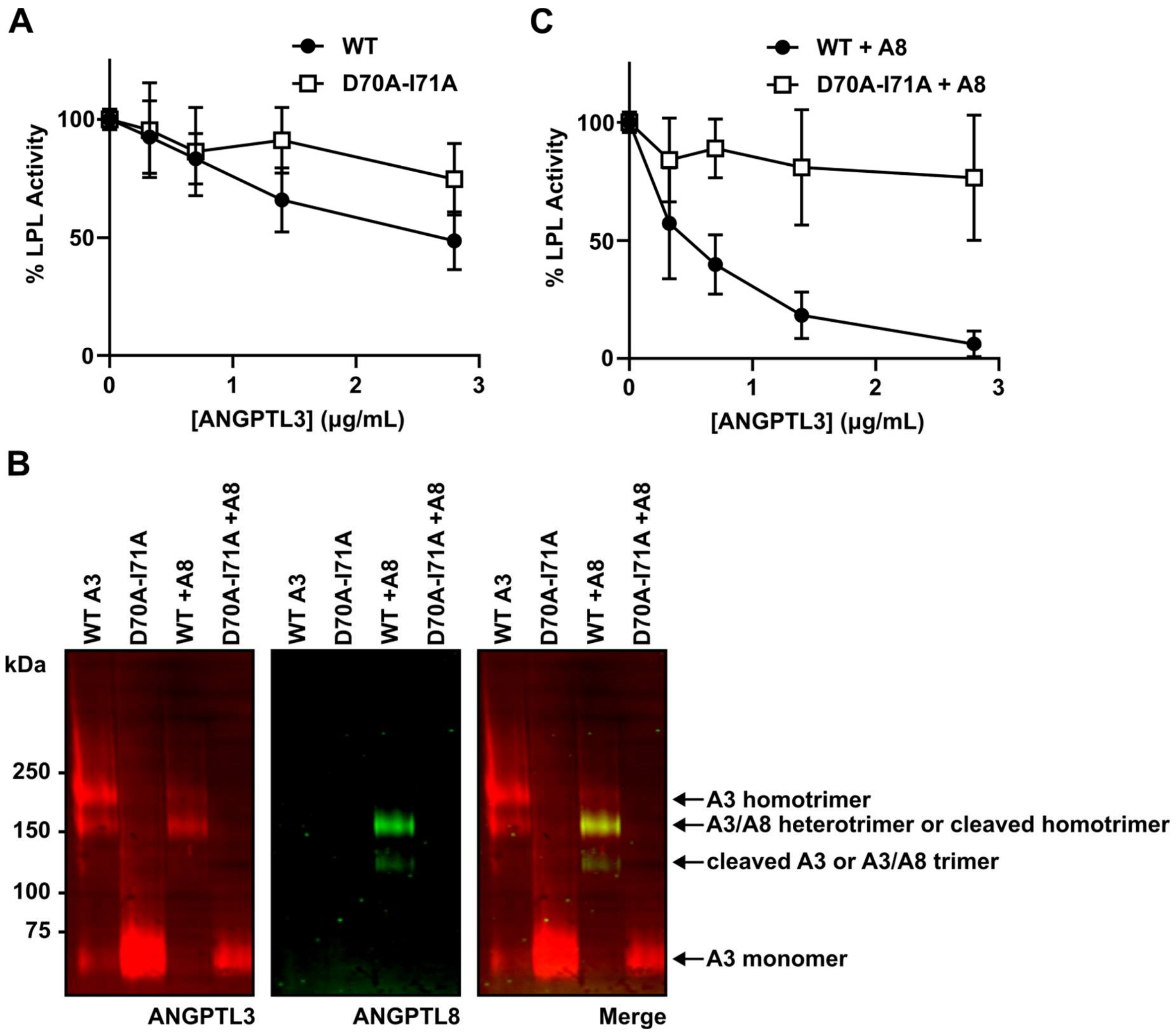

Supplemental Figure 6: Inhibition of LPL by mutant ANGPTL3. (A) Triglyceride lipase activity of LPL after incubation with the indicated concentrations of wild-type ANGPTL3 or ANGPTL3 D70A-I71A. LPL and ANGPTL3 were incubated for 30 minutes at 37ºC and then triglyceride activity was measured using a fluorescence-based assay. Points represent the mean (±SD) of three independent experiments performed in duplicate. For each graph, the activity was normalized to the control treated with LPL and 0 µg/ml ANGPTL3. **(B)** Conditioned media containing wild-type and mutant mouse ANGPTL3 expressed with and without ANGPTL8 (A8) were run on blue nativePAGE gels and blotted for ANGPTL3 (red) and ANGPTL8 (green). **(C)** Triglyceride lipase activity of LPL after incubation with the indicated concentrations of wild-type ANGPTL3 or ANGPTL3 D70A-I71A, both co-expressed with ANGPTL8. LPL and ANGPTL3/8 were incubated for 30 minutes at 37ºC and then triglyceride activity was measured using a fluorescence-based assay. Points represent the mean ± SD of three independent experiments performed in duplicate. For each graph, the activity was normalized to the control treated with LPL and 0 µg/ml ANGPTL3/8.
