# Supplementary material for: Angiopoietin-like 3 monomers are abundant in human plasma but are unable to inhibit endothelial lipase
Source: JCI Insight. 2025 Oct 28;10(23):e197827. doi: 10.1172/jci.insight.197827 (PMC12867014; doi:10.1172/jci.insight.197827)

Full unedited gel for Figure 1A

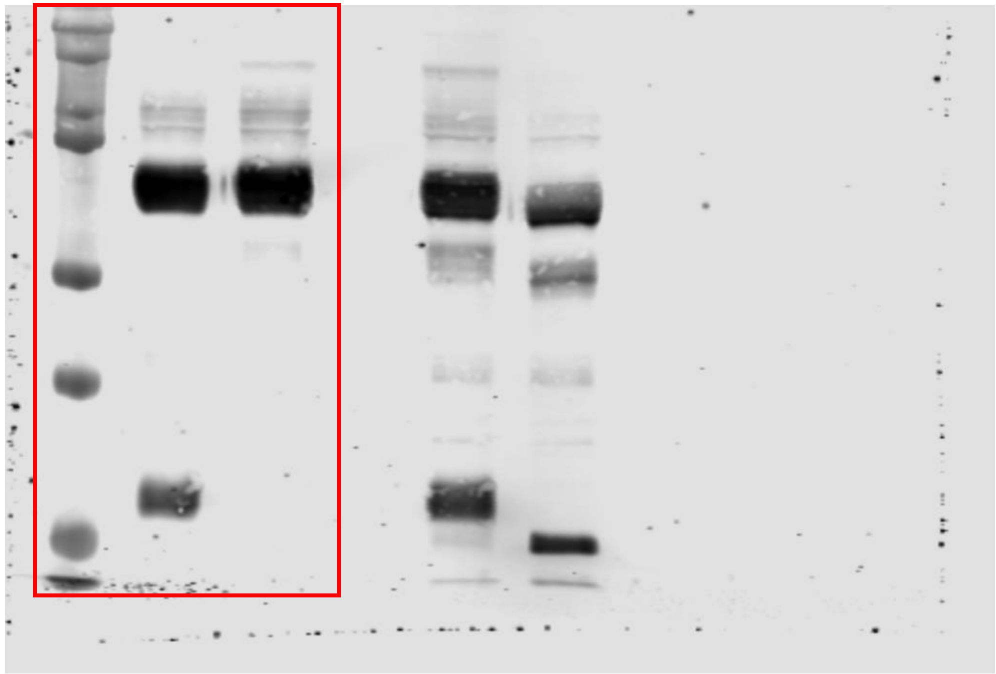

Full unedited gel for Figure 1B

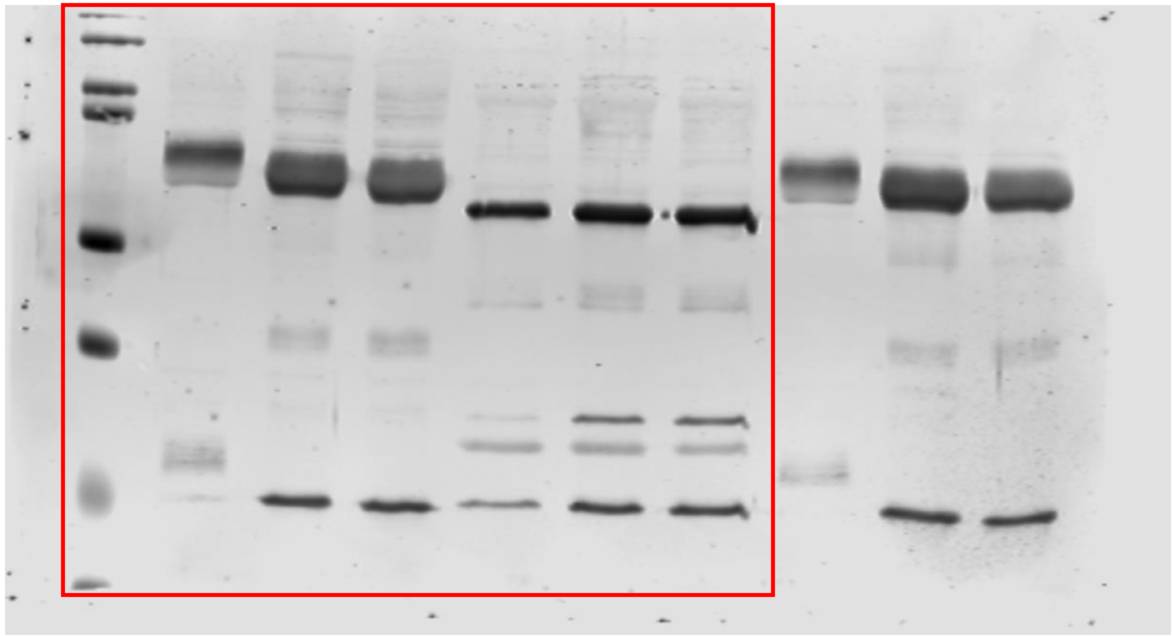

Full unedited gel for Figure 4A

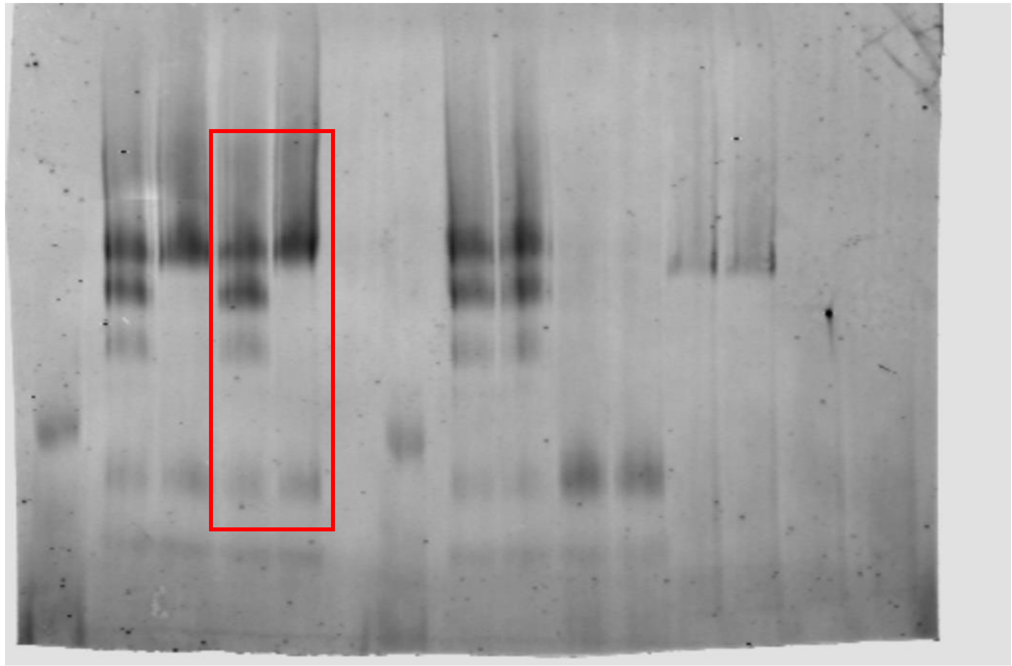

Full unedited gel for Figure 4B

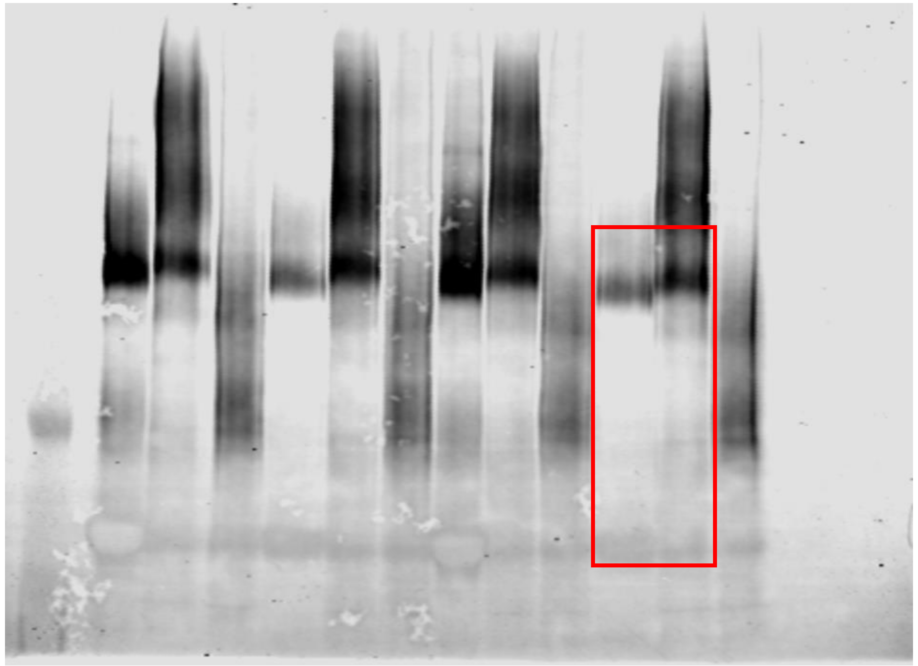

Full unedited gel for Figure 5B

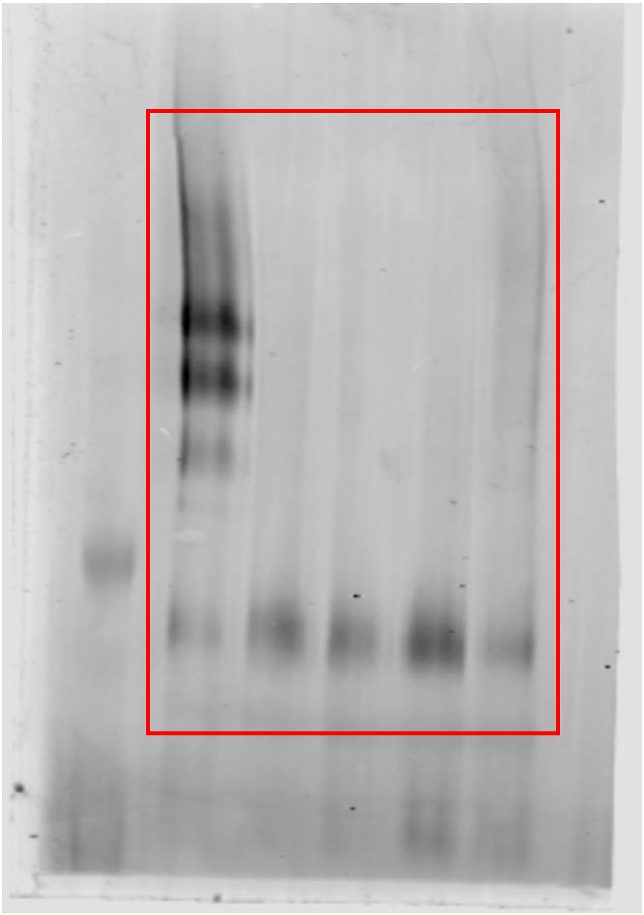

Full unedited gel for Figure 6B

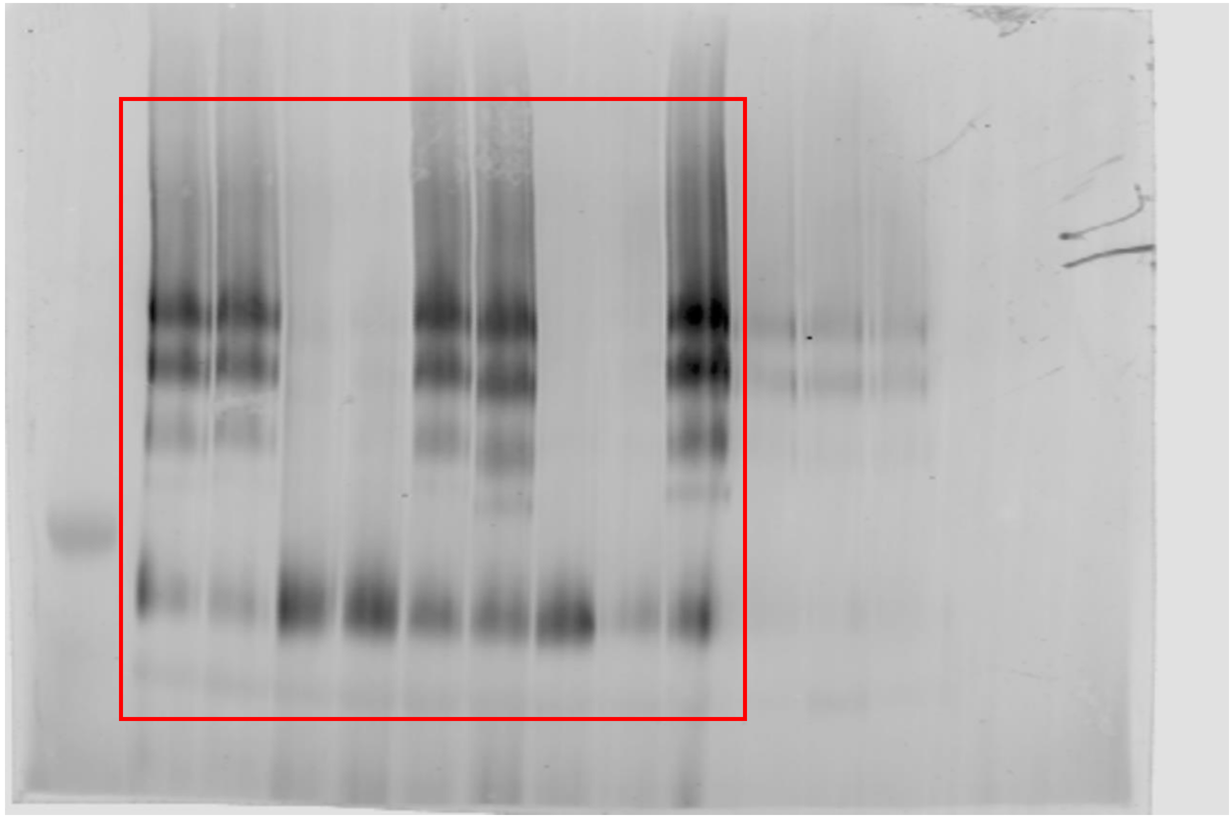

Full unedited gel for Figure 7C

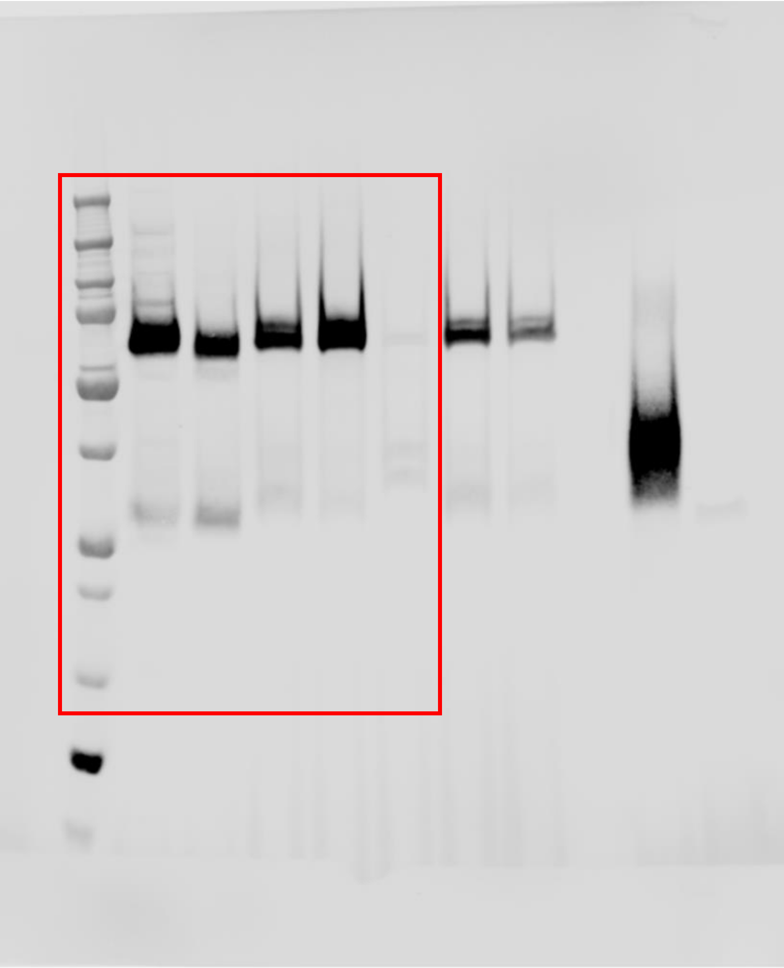

Full unedited gel for Figure 7D

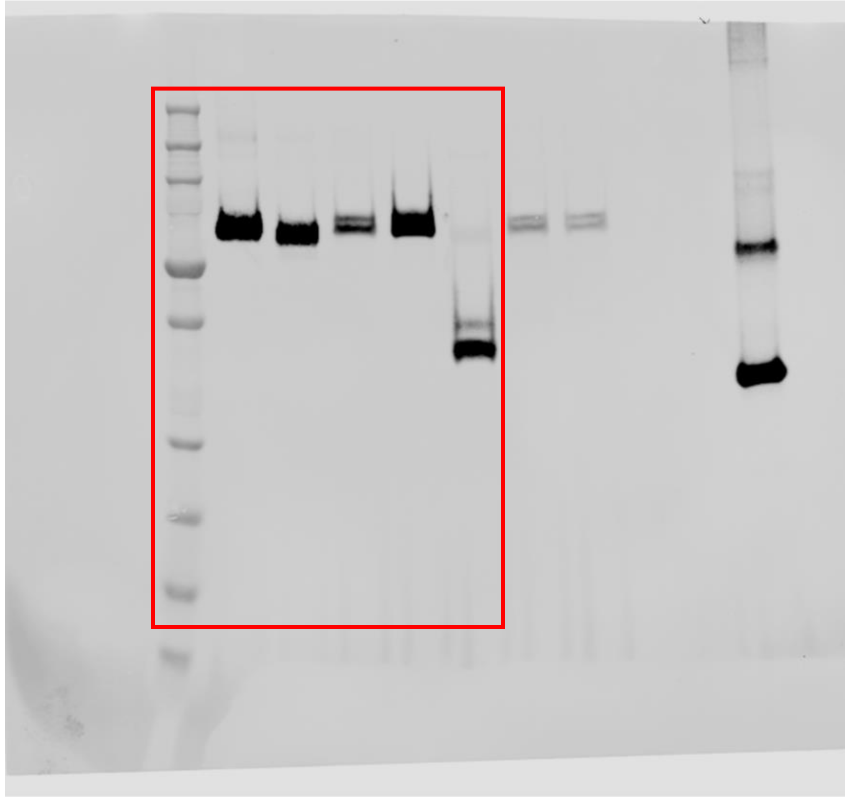

Full unedited gel for Figure 8A

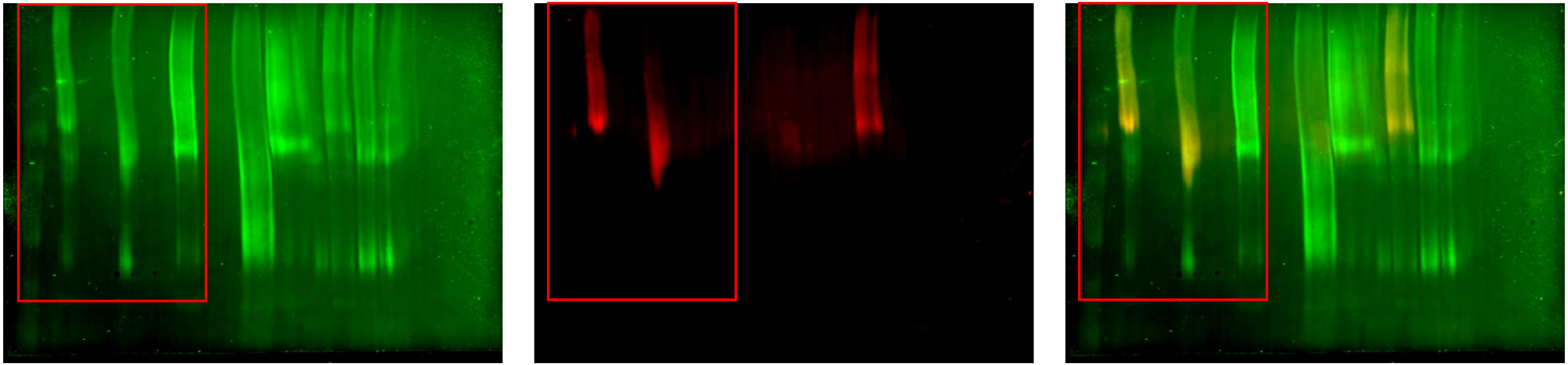

Supplement: Unedited blot and gel images [file jciinsight-10-197827-s267.pdf]
